# Exploration of efficient electron acceptors for organic solar cells: rational design of indacenodithiophene based non-fullerene compounds

**DOI:** 10.1038/s41598-021-99254-4

**Published:** 2021-10-07

**Authors:** Muhammad Khalid, Muhammad Usman Khan, Eisha-tul -Razia, Zahid Shafiq, Mohammed Mujahid Alam, Muhammad Imran, Muhammad Safwan Akram

**Affiliations:** 1 Department of Chemistry, Khwaja Fareed University of Engineering and Information Technology, Rahim Yar Khan, 64200 Pakistan; 2 Department of Chemistry, University of Okara, Okara, 56300 Pakistan; 3 Institute of Chemical Sciences, Bahauddin Zakariya University, Multan, 60800 Pakistan; 4 Department of Chemistry, Faculty of Science, King Khalid University, P.O. Box 9004, Abha, 61413 Saudi Arabia; 5 School of Health and Life Sciences, Teesside University, Middlesbrough, TS1 3BX UK; 6 National Horizons Centre, Teesside University, Darlington, DL1 1HG UK

**Keywords:** Computational chemistry, Density functional theory

## Abstract

The global need for renewable sources of energy has compelled researchers to explore new sources and improve the efficiency of the existing technologies. Solar energy is considered to be one of the best options to resolve climate and energy crises because of its long-term stability and pollution free energy production. Herein, we have synthesized a small acceptor compound (**TPDR**) and have utilized for rational designing of non-fullerene chromophores (**TPD1**–**TPD6**) using end-capped manipulation in A2–A1–D–A1–A2 configuration. The quantum chemical study (DFT/TD-DFT) was used to characterize the effect of end group redistribution through frontier molecular orbital (FMO), optical absorption, reorganization energy, open circuit voltage (*Voc*), photovoltaic properties and intermolecular charge transfer for the designed compounds. FMO data exhibited that **TPD5** had the least ΔE (1.71 eV) with highest maximum absorption (*λ*_*max*_) among all compounds due to the four cyano groups as the end-capped acceptor moieties. The reorganization energies of **TPD1**–**TPD6** hinted at credible electron transportation due to the lower values of *λ*_*e*_ than *λ*_*h*_. Furthermore, open circuit voltage (*Voc*) values showed similar amplitude for all compounds including parent chromophore, except **TPD4** and **TPD5** compounds. These designed compounds with unique end group acceptors have the potential to be used as novel fabrication materials for energy devices.

## Introduction

In the past century, fossil fuels including natural gas, petroleum products and coal have been utilized all over the world to produce electricity^1^. However, with the passage of time due to the detrimental effect on environment, dependency on fossil fuels is decreasing. Nevertheless, when it comes to meet the green targets, almost all governments are struggling to meet the required limits of CO_2_ emissions. Therefore, it is a dire need to find environment friendly yet efficient renewable energy sources from the current mix of wind power^2^, biomass, hydro power^3^, and solar cells which have shown promise for energy generation^4,5^. It comes as no surprise, that solar energy is one of the fastest growing energy technologies today, with the rise of solar parks around the globe and deployment of photovoltaic (PV) cells on domestic roof tops. The leading PVs are silicon-based solar cells (SBSCs), which have been used for the large scale production of electrical energy due to relatively high power conversion efficiencies (PCEs), large natural abundance and high thermal stability. The drawbacks of SBSCs include non-tunable energy levels, high cost, brittleness, heavy weight (20–30 kg m^−2^) to the extent that it has limited use of these solar cells on modern curved buildings. Most of these challenges can be overcome by the use of organic solar cells^6,7^ (OSCs) which are more flexible and light weight (0.5 kg m^−2^). OSCs are bulk heterojunction (BHJ) devices where donor and acceptor moieties are mixed together in the absorption layer, where absorbance spectrum ideally should match the solar spectrum to make most of the falling sun light. Historically OSCs have suffered from low PCEs which was changed by the use of fullerene derivatives that showed much better PCEs because of isotropic charge transfer (ICT), high electron mobility and low reorganization energy values^8,9^. However, fullerene compounds suffer from poor solubility, instability and high manufacturing cost^10–13^. Furthermore, glass-forming small non-fullerene donor or acceptor materials creating interfacial layer show significant importance in the development of OSCs^14–17^. Therefore, non-fullerene electron acceptors (NFAs) have gained attraction^18^ due to their remarkable visible region absorption, good solubility, better stability, easy tuning of energy levels and relative low cost^19–21^.

Literature is replete with solar cells based on small molecules having acceptors and donors arranged various geometries such as X-shaped donor molecule^22^, star molecule^23^ and linear geometric molecules^24^, etc. In these molecules, the extended π-conjugation responsible for absorbance of non-fullerene have been enhanced via attachment of fluoro^25,26^, chloro and cyano groups over the skeleton structure. One if the most successful arrangement, is D–$\pi$–A architecture, which allows transfer of electrons from donor (D) to acceptor (A) via π-bridge^27–29^. In this manuscript, we have taken this established architecture A_2_–A_1_–D–A_1_–A_2_ to further explore the potential of thieno[3,4-c]pyrrole-4,6-dione (**TPD**). This reference chromophore (**TPDR**^30^) consists of 2-(1,1-dicyanomethylene)-rhodamine (**RCN**), thieno[3,4-c]pyrrole-4,6-dione (**TPD**), and indacenodithiophene (**IDT**) are A2, A1, and D moieties, respectively. Further, six novel molecules (**TPD1**–**TPD6**) are in-silico designed from **TPDR** by modifying end-capped acceptor units. To the best of our information, the photovoltaic investigation of these designed compounds is unreported. Herein we report absorption maxima (*λ*_*max*_), frontier molecular orbital (FMOs) and density of states (DOS) analysis, open-circuit voltage (*Voc*), reorganization energies and transition density matrix (TDM) of **TPD1**–**TPD6** and have drawn comparison with the reference **TPDR** to evaluate the performance of end-capped acceptor units.

## Results and discussion

**Figure 1:**
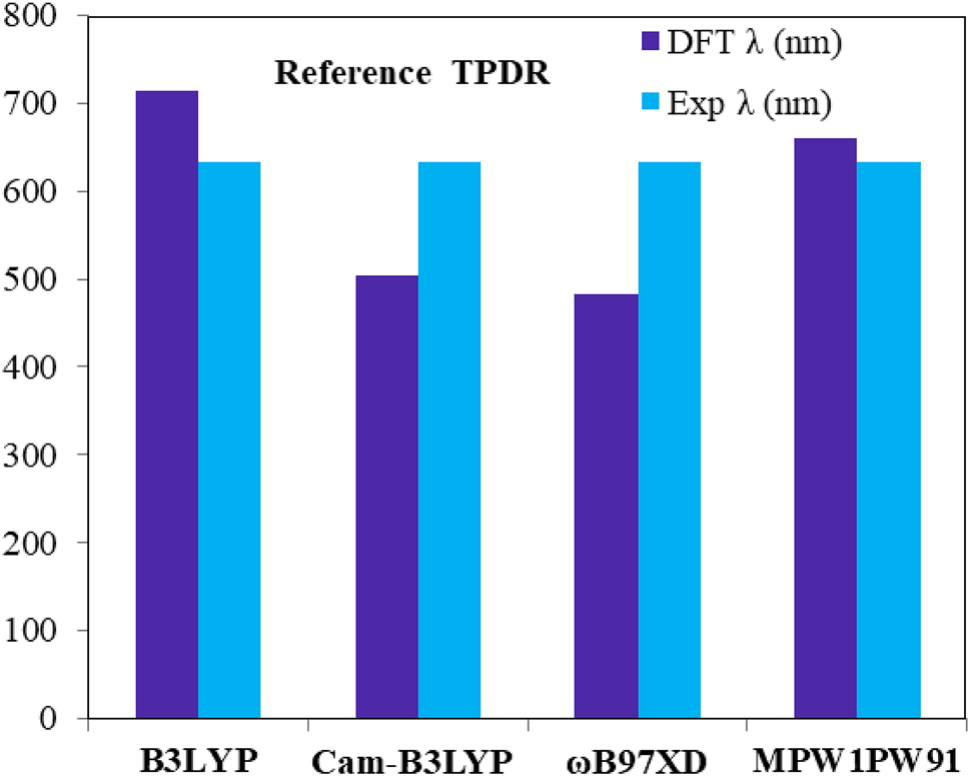
The simulated UV–vis results of **TPDR** at different DFT functionals.

**Figure 2:**
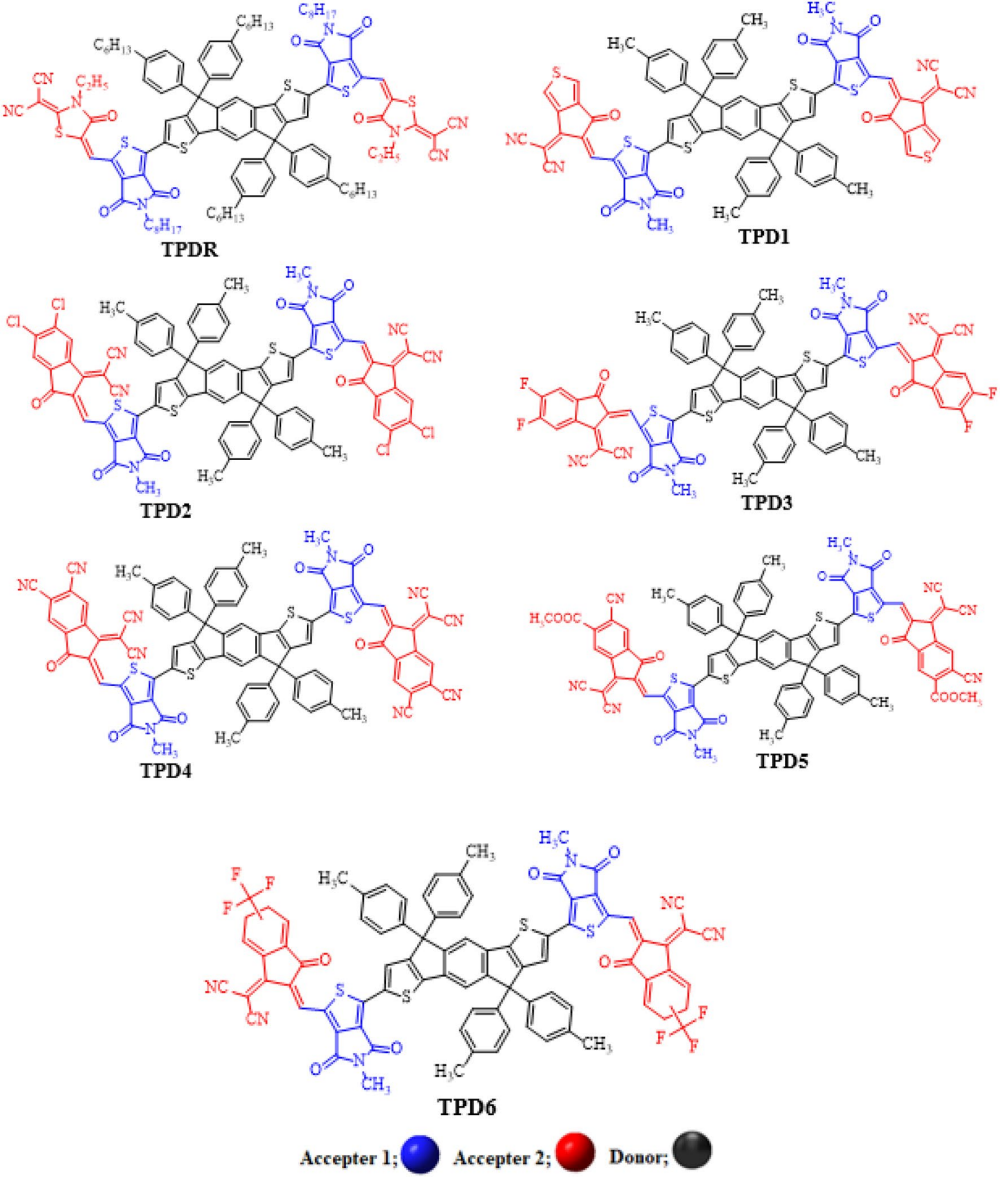
The 2D-structures of entitled compounds for clear view. 2,2′-((5Z,5′Z)-(((4,4,9,9-tetra-p-tolyl-4,9-dihydro-s-indaceno[1,2-b:5,6-b′]dithiophene-2,7-diyl)bis(5-methyl-4,6-dioxo-5,6-dihydro-4H-thieno[3,4-c] pyrrole-3,1diyl))bis (methaneylylidene))bis(6-oxo-5,6-dihydro-4H-cyclopenta[c]thiophene-5,4-diyli idene))dimalononitrile (**TPD1**), 2,2′-((2Z,2′Z)-(((4,4,9,9-tetra-p-tolyl-4,9-dihydro-s-indaceno[1,2-b:5,6-b′]dithiophene-2,7-diyl)bis(5-methyl-4,6-dioxo-5,6-dihydro-4H-thieno[3,4-c]pyrrole-3,1-diyl) )bis(methaneyly-lidene))bis(5,6-dichloro-3-oxo-2,3-dihydro-1H-indene-2,1-diylidene))dimalono- nitrile (**TPD2**), 2,2′-((2Z,2′Z)-(((4,4,9,9-tetra-p-tolyl-4,9-dihydro-s-indaceno[1,2-b:5,6-b′]dithio phene-2,7-diyl)bis(5-methyl-4,6-dioxo-5,6-dihydro-4H-thieno[3,4-c]pyrrole-3,1-diyl))bis(methane ylylidene))bis(5,6-difluoro-3-oxo-2,3-dihydro-1H-indene-2,1-diylidene))dimalononitrile (**TPD3**), (2Z,2′Z)-2,2′-(((4,4,9,9-tetra-p-tolyl-4,9-dihydro-s-indaceno[1,2-b:5,6-b′]dithiophene-2,7-diyl)bis(5-methyl-4,6-dioxo-5,6-dihydro-4H-thieno[3,4-c]pyrrole-3,1-diyl))bis(methaneylylidene))bis(1-(dicyanomethyl ene)-3-oxo-2,3-dihydro-1H-indene-5,6-dicarbonitrile) (**TPD4**), methyl (Z)-6-cyano-2-((3-(7-(3-(((Z)-5-cyano-1-(dicyanomethylene)-6-(methoxycarbonyl)-3-oxo-1,3-dihydro-2H-inden-2-ylidene)methyl)-5-methyl-4,6-dioxo-5,6-dihydro-4H-thieno[3,4-c]pyrrol-1-yl)-4,4,9,9-tetra-p-tolyl-4,9-dihydro-s-indaceno[1,2-b:5,6-b′]dithiophen-2-yl)-5-methyl-4,6-dioxo-5,6-dihydro-4H-thieno[3,4-c]pyrrol-1-yl)methylene)-1-(dicyanomethylene)-3-oxo-2,3-dihydro-1H-indene-5-carboxylate (**TPD5**) and 2,2′-((2Z,2′Z)-(((4,4,9,9-tetra-p-tolyl-4,9-dihydro-s-indaceno[1,2-b:5,6-b′]dithiophene-2,7-diyl)bis(5-methyl-4,6-dioxo-5,6-dihydro-4H-thieno[3,4-c]pyrrole-3,1-diyl)) bis(methaneylylidene))bis(3-oxo-2,3,5,6-tetrahydro-1H-indene-2,1-diylidene))dimalononitrile (**TPD6**).

**Figure 3:**
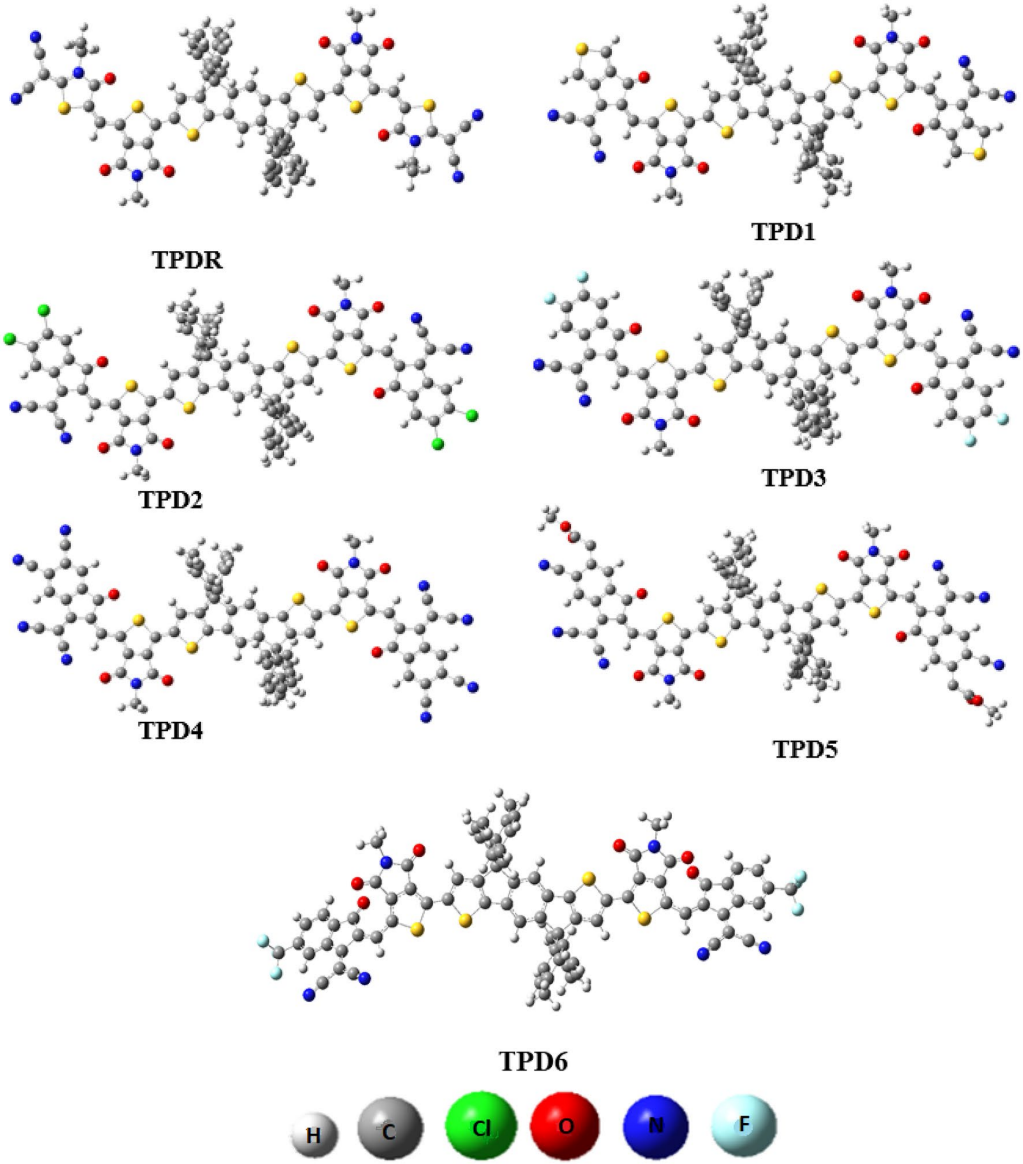
The optimized structures of **TPDR** and **TPD1**–**TPD6** at MPW1PW91 6-31G(d,p). Figures are made with are made with the help of GaussView 5.0 and Gaussian 09 version D.01 (https://gaussian.com/g09citation/).

The absorption maxima of the reference chromophore, synthesized **TPDR** NFA by Li et al.^30^ was investigated in chloroform at four functionals: MPW1PW91, ωB97XD , B3LYP and CAM-B3LYP in conjunction with 6-31G (d,p) basis set and *λ*_*max*_ of **TPDR** was calculated to be 714.15, 503.63, 483.12 and 658.89 nm, respectively. The functional, MPW1PW91/6-31G(d,p) showed the best agreement between the computed and experimental values at 632 nm as shown in Fig. 1. Therefore, this *λ*_*max*_ value was most appropriate for further investigation on our designed compound**s**. The terminal acceptor groups of **TPDR** were substituted sequentially with different acceptor unit as shown in Figure S1 to design efficient non-fullerene OSCs. By replacing end-capped acceptors, six distinct derivatives namely **TPD1, TPD2, TPD3, TPD4, TPD5** and **TPD6** were obtained, their IUPAC names and two-dimensional (2D) structures are presented in Fig. 2 while their optimized molecular geometries of investigated compounds are presented in Fig. 3.

### Frontier molecular orbitals (FMOs) analysis

FMOs analysis helps to understand the intramolecular charge transfer (ICT) characteristics, optoelectronic properties and electron density distribution of chromophores^31–37^ by estimating the charge transition between LUMO and HOMO orbitals^38^. Band theory describes the LUMO and HOMO as valence and conduction bands, respectively. The energy difference among orbitals is explained as bandgap (*ΔE*)^39,40^. The performance of OSCs can be explained with the help of *ΔE or Eg*, as there will be greater power conversion efficiency (PEC) of a photovoltaic material with lower bandgap and *vice versa*^41–43^. Herein, we analyze the conducting behavior of electronic density accompanying photon characteristics of the designed compounds. Data in Table 1 shows energies of orbitals and bandgaps of **TPDR** and **TPD1**–**TPD6**.

**Table 1:** The E_HOMO_, E_LUMO_ and *ΔE *(E_LUMO_ − E_HOMO_) of entitled chromophores.

Compounds	E_HOMO_	E_LUMO_	*ΔE*
**TPDR**	− 5.71	− 3.38	2.33
**TPD1**	− 5.70	− 3.58	2.12
**TPD2**	− 5.80	− 3.71	2.10
**TPD3**	− 5.77	− 3.64	2.13
**TPD4**	− 6.05	− 4.09	1.96
**TPD5**	− 5.99	− 4.28	1.71
**TPD6**	− 5.79	− 3.68	2.11

Units in eV*.*

For **TPDR**, energies for HOMO/LUMO are measured as − 5.71 and − 3.38 eV with 2.33 eV of energy gap. Theoretically calculated HOMO/LUMO energies are found to − 5.48 and − 3.42 eV*, which are* in close agreement with the experimental values^30^. The end-capped acceptor units of **TPDR** has been replaced with powerful electron-withdrawing groups and subsequently, a reduction in band gap is observed in all the derivatives as seen in Table 1.

The descending order for HOMO/LUMO energies of designed chromophores is noted to be **TPD1** > **TPDR** > **TPD3** > **TPD6** > **TPD2** > **TPD5** > **TPD4** and **TPDR** > **TPD1** > **TPD3** > **TPD6** > **TPD2** > **TPD4** > **TPD5**, respectively. Overall, reduction in the bandgap is noted particularly in TDP3 where terminal acceptor unit is modified with 2-(5,6-difluoro-2-methylene-3-oxo-2,3-dihydro-1H-inden-1-ylidene)malononitrile which leads to extended conjugation combined with the introduction of powerful electron-pulling units together lowers the E_g_. Similar reductions in bandgap were observed at 2.13, 2.12, 2.11 and 2.10 eV, respectively is for **TPD3**, **TPD1**, **TPD6** and **TPD2**. Even further decrease of E_g_ is observed in **TPD4** and **TPD5** at 1.96 and 1.71 eV, respectively where end-capped acceptors are changed to 1-(dicyanomethylene)-2-methylene-3-oxo-2,3-dihydro-1H-indene-5,6-dicarbonitrile and methyl 6-cyano-3-(dicyanomethylene)-2-methylene-1-oxo-2,3-dihydro-1H-indene-5-carboxylate, respectively. This decrease in E_g_ can be attributed to the introduction of cyano (-CN) units^44^. Consequently, least bandgap among all the chromophores has been exhibited in **TPD5**, as the powerful pulling nature of cyano and ester group on a conjugation stabilized chromophore. Overall, the descending order for E_g_ of entitled chromophores is found to be **TPDR** > **TPD3** > **TPD1** > **TPD6** > **TPD2** > **TPD4** > **TPD5**.

**Figure 4:**
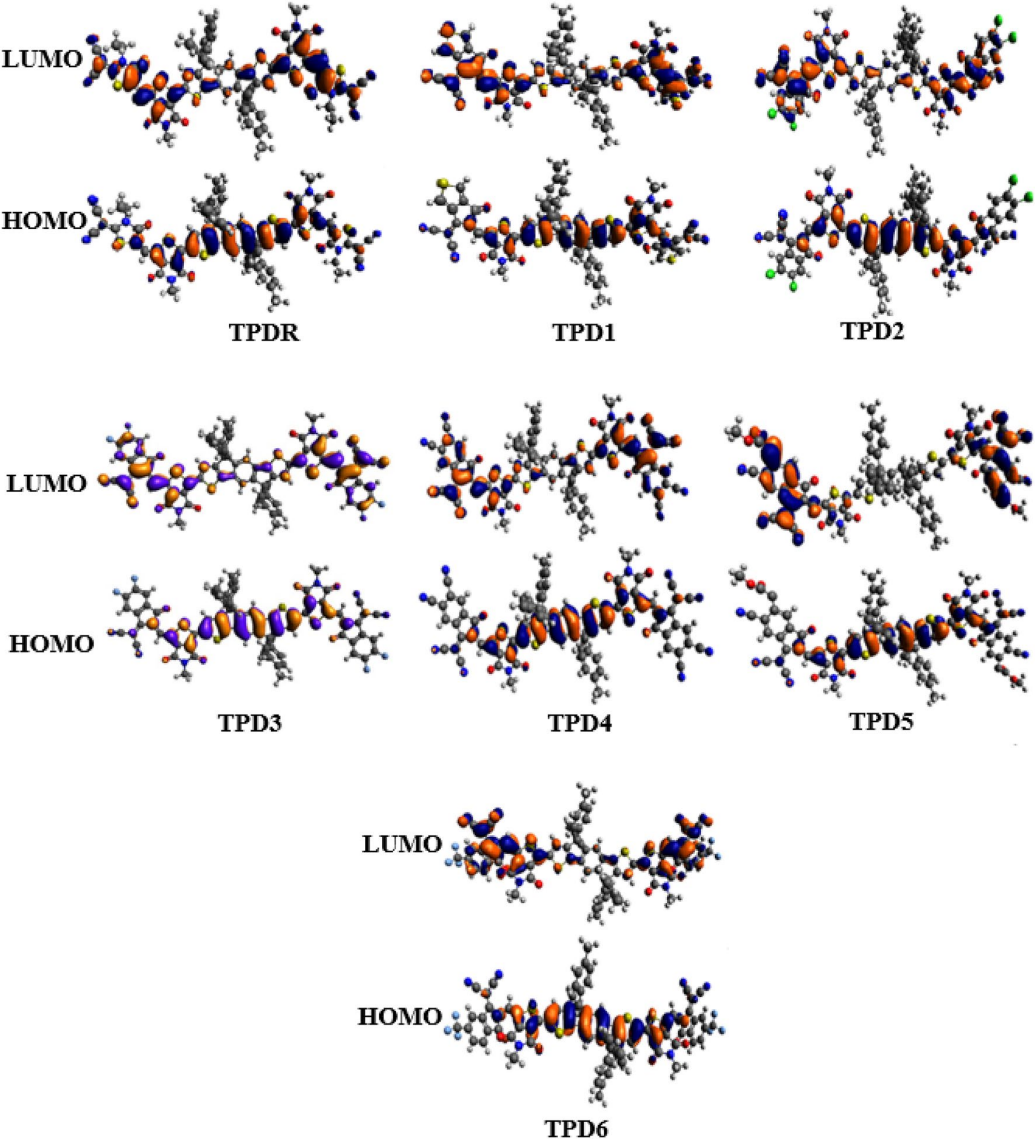
HOMOs and LUMOs of **TPDR** and **TPD1**–**TPD6**.

In terms of charge transfer, chromophores with lesser E_g_ possessed greater charge transfer rate and henceforth, demonstrated larger photovoltaic response^45^. The electronic charge density for HOMO is located significantly over the central donor unit except the methyl groups and A1 unit in most of the designed chromophores. Similarly, for LUMO charge density is concentrated dominantly over the acceptors (A1 and A2) and for a minor part on the central donor moiety (Fig. 4). Overall, all the designed molecules showed significant charge transfer between orbitals indicating their potential to be good photovoltaic materials.

### Density of states

**Figure 5:**
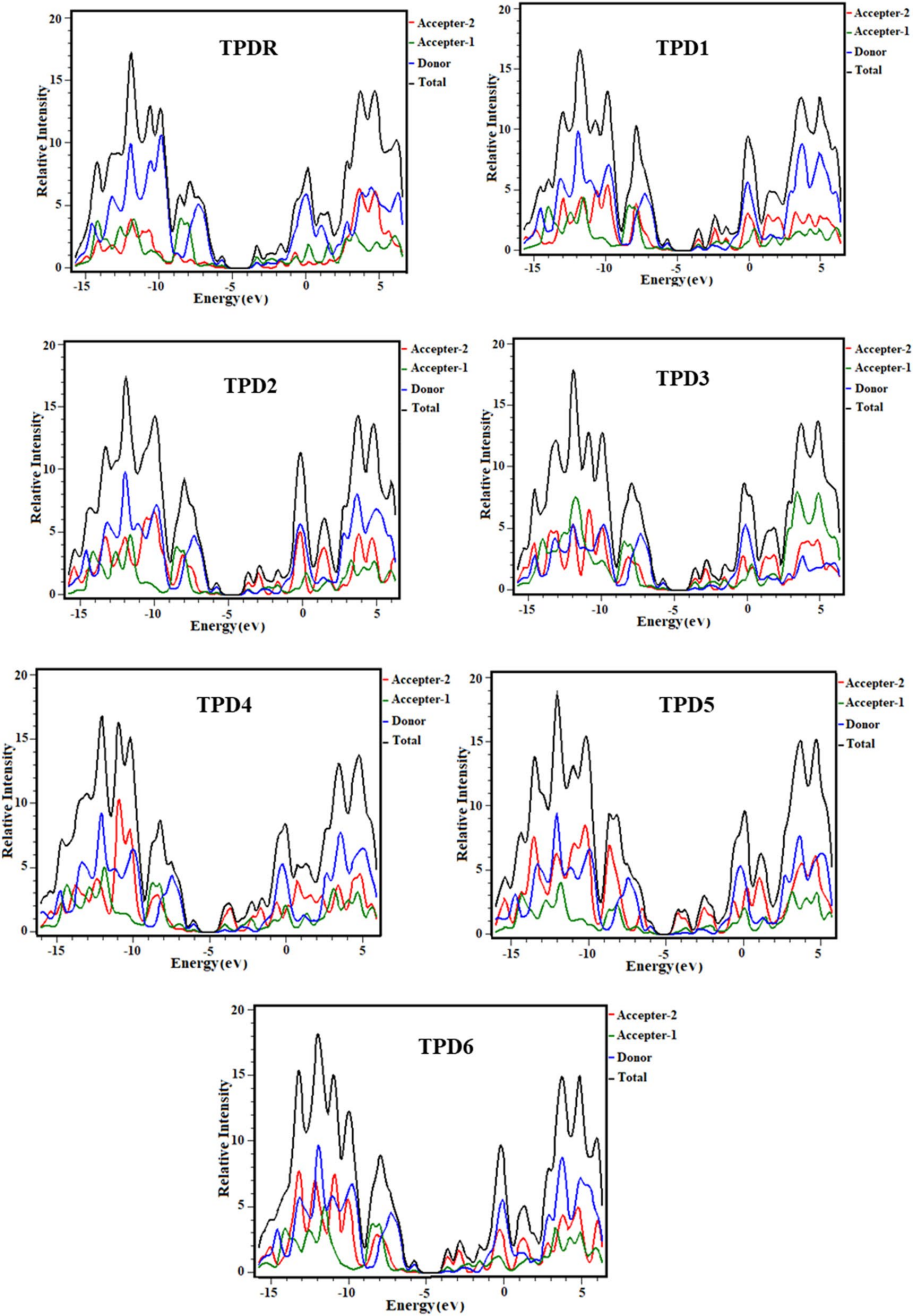
Graphical representation of the density of states (DOS) of studied chromophores drawn by utilizing PyMOlyze 1.1 version (https://sourceforge.net/projects/pymolyze/). All out put files of entitled compounds were computed through Gaussian 09 version D.01 (https://gaussian.com/g09citation/).

The DOS analysis of **TPD1**–**TPD6** and **TPDR** are executed using MPW1PW91/6-31G(d,p) functional and DOS spectra are portrayed in Fig. 5.

**Figure 6:**
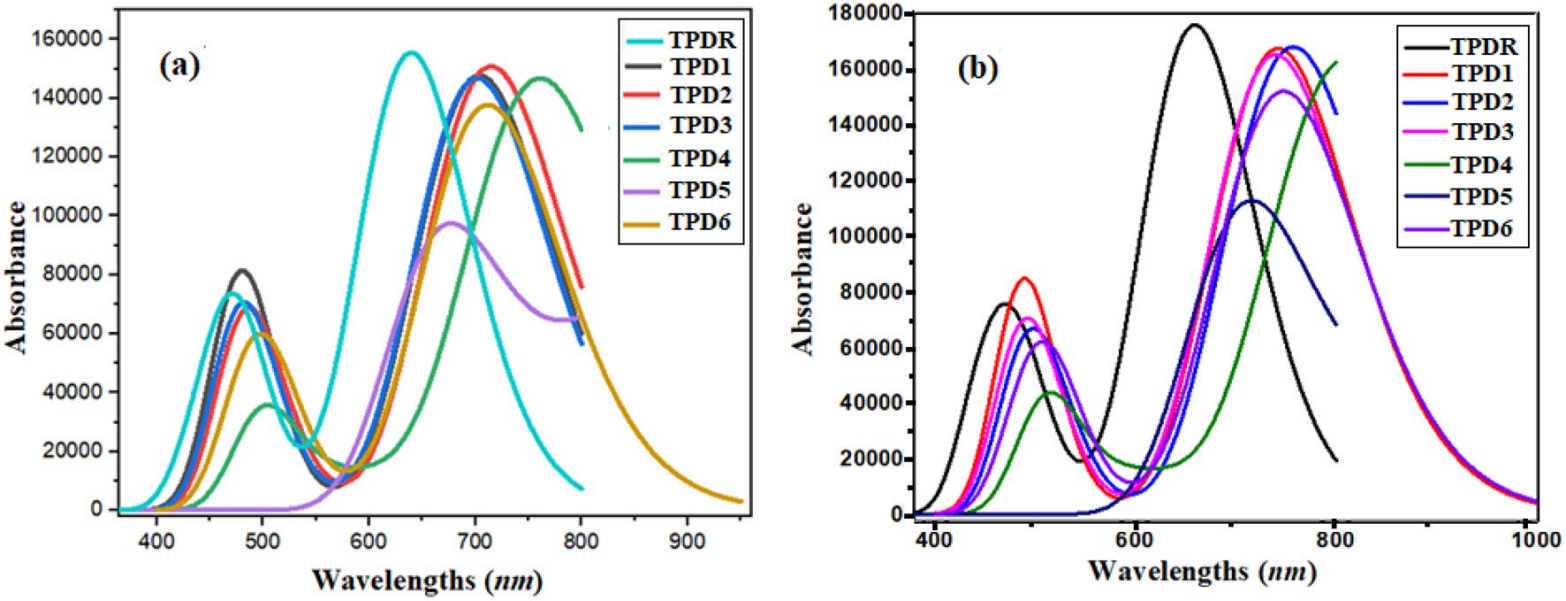
Simulated absorption spectra (**a**) in gaseous phase and (**b**) in chloroform of studied compounds.

For DOS analysis, we have divided our compounds into three portions, which are Donor (core unit), Acceptor 1 (bridge) and Acceptor 2 (end-capped acceptor group) represented by blue, green and red lines, respectively in Fig. 5. The negative values show the HOMO (valence band) while positive values express LUMO (conduction band) along x-axis and distance between both conduction band and valence band represents the band gap. For **TPDR** the Acceptor-1 contributes 22.3% to HOMO and 48.8% to LUMO, while Acceptor-2 contributes 11.4% to HOMO and 35.6% to LUMO. Similarly, Donor contributes 66.4% to HOMO and 15.6% to LUMO in **TPDR**. The Acceptor-1 contributes 20.4%, 20.1%, 21.0%, 19.4%, 9.4%, and 21.0% to HOMO and 28.6%, 28.1%, 29.8%, 25.6%, 19.1% and 24.4% to LUMO in **TPD1**–**TPD6**, respectively. Similarly, Acceptor-2 contributes 11.7%, 11.5%, 10.5%, 12.5%, 12.7% and 9.9% to HOMO, while 59.4%, 60.1%, 58.1%, 64.0%, 77.3% and 65.2% to LUMO for **TPD1**–**TPD6**, respectively. In the same way, donor contributes 68.0%, 68.3%, 68.5%, 68.1%, 77.9% and 69.2% to HOMO, and 12.0%, 11.8%, 12.1%, 10.4%, 3.6%, and 10.4% to LUMO for **TPD1**–**TPD6**, separately. By these findings, it is examined from DOS graphs that the HOMOs are largely concentrated on donor as higher peak of blue color which is located nearly -5.6 eV*.* Similarly, the LUMOs are significantly on A_1_ in **TPDR** while on A_2_ in all derivatives as higher peak is located near 6.5 eV hence, these graphs significantly support the FMO diagrams (see Figs. 5, 6). Overall, the charge density circulation reveals that significant amount of charge is relocated due to delocalization of electrons in case of **TPDR** and all its derivatives from the central D to end-capped A units with the assistance of the Acceptor 1.

### UV–visible analysis

TD-DFT investigations are utilized to find UV–Vis spectra at MPW1PW91/6-31G(d, p) functional to elucidate optoelectronic properties for entitled chromophores.

The studied compounds are of A_2_–A_1_–D–A_1_–A_2_ type with different end-capped acceptors leading to differing optoelectronic responses. In all of the investigated compounds higher *λ*_*max*_ and low transition energy values are observed in both gas and chloroform solvent. The significant oscillator strength (*f*_*os*_), excitation energy and maximum absorption *λ*_*max*_ in gas and chloroform are presented in Tables 2 and 3 while other transitions are shown in Tables S8–S21 and their absorption spectra is shown in Fig. 6. The outcomes illustrate the greater red shift in *λ*_*max*_ of novel compounds due to strong electron-withdrawing units at end-capped terminal moiety with extended conjugation. **TPD1**–**TPD6** compounds give higher red shift along lesser excitation energy contrasted with **TPDR**.

**Table 2:** Wavelength (*λ*_*max*_), excitation energy (E) and oscillator strength (*f*_*os*_) of designed compounds in gas.

Compounds	λ (nm)	E (eV)	*f*	MO contributions
**TPDR**	639.29	1.94	2.14	H → L (97%)
**TPD1**	703.72	1.76	2.04	H → L (98%)
**TPD2**	714.65	1.73	2.08	H → L (97%)
**TPD3**	701.34	1.77	2.03	H → L (97%)
**TPD4**	761.15	1.63	2.02	H → L (98%)
**TPD5**	673.53	1.84	1.32	H → L + 2 (97%)
**TPD6**	712.31	1.74	1.88	H → L (97%)

*MO* molecular orbital, *H* HOMO, *L* LUMO.

**Table 3:** Wavelength (*λ*_*max*_), excitation energy (***E***) and oscillator strength (***f***_***os***_) of investigated compounds in chloroform.

Compounds	*λ*_*max*_ (nm)	E (eV)	*f*_*os*_	MO contributions
**TPDR**	658.89 (632)^a^	1.88	2.42	H → L (95%)
**TPD1**	741.93	1.67	2.31	H → L (96%)
**TPD2**	757.11	1.63	2.31	H → L (96%)
**TPD3**	739.63	1.67	2.27	H → L (96%)
**TPD4**	810.78	1.53	2.25	H → L (96%)
**TPD5**	714.56	1.73	1.54	H → L + 2 (96%)
**TPD6**	748.65	1.65	2.05	H → L (95%),

*MO* molecular orbital, *H* HOMO, *L* LUMO.

^a^Exp. value in parentheses^30^.

The absorption of all the studied compounds is located in the range of 658.89–810.78 nm in chloroform and 639.29–761.15 nm in gas. Interestingly, it was observed that all the chromophores show red shift in chloroform than in the gaseous phase except for **TPD4**, which expressed higher absorption wavelength (810.78 nm) in chloroform than in the gas phase (761.15 nm). This might be owing to the interaction of cyano unit on the terminal acceptor with chloroform which stabilized the molecule.

Table 3 reveals that the *λ*_*max*_ calculated for **TPDR** is 658.89 nm in chloroform, which correlates well with the experimental value (632 nm). Owing to the solvent effect, observed *λ*_*max*_ results in chloroform as red shifted in comparison to while dissolved in gaseous phase. The *λ*_*max*_ order is found to be **TPDR** < **TPD5** < **TPD3** < **TPD1** < **TPD6** < **TPD2** < **TPD4**. The lower excitation energies of all new designed compounds depicted the easy excitation between HOMO and LUMO in contrast to **TPDR**. The excitation energy (*E*) increasing order is obtained to be **TPD4** < **TPD2** < **TPD6** < **TPD3** = **TPD1** < **TPD5** < **TPDR**. This confirms that the designed non-fullerene acceptor compounds (**TPD1**–**TPD6**) have enhanced optical properties than **TPDR**.

### Reorganization energy

The hole and electron reorganization energy (RE) are considered as a fundamental tool to estimate the performance and working capability of OSCs. Reorganization energy is inversely related to the charge mobility. Chromophores with least RE exhibit greater mobilities of hole and electron or *vice versa*^46^. Reorganization energy depends upon myriad factors amongst which geometric shape of cations and anions has the major influence. The cationic geometry displays the hole while anionic geometry shows the electron transportation towards acceptor from donor molecule.

The reorganization energy has two major categories: *λ*_*ext*_*.* responds to exterior environmental changes and *λ*_*int*_. denotes internal reorganization energy and provides information for the internal structural rapid changes. Herein, we ignored the external environmental influence as it does have minimal impact. RE is calculated by utilizing Eqs. (3) and (4) to understand charge mobility of **TPDR** and **TPD1**–**TPD6** chromophores, and results are displayed in Table 4.

**Table 4:** Reorganization energies ($\lambda_{e}$; rate of transfer of electrons*;* $\lambda_{h}$: rate of transfer of hole) of studied molecules.

Compounds	$\lambda_{e}$ (eV)	$\lambda_{h}$ (eV)
**TPDR**	0.00736	0.00342
**TPD1**	0.00528	0.00681
**TPD2**	0.00547	0.00697
**TPD3**	0.00577	0.00701
**TPD4**	0.00433	0.00679
**TPD5**	0.00904	0.00789
**TPD6**	0.00661	0.00785

The calculated reorganization energy of electron (*λ*_*e*_) for **TPDR** and **TPD1**–**TPD6** were 0.00736, 0.00528, 0.00547, 0.00577, 0.00433, 0.00904 and 0.00661 eV, respectively. All derivatives showed value of *λe* in the range of 0.00433–0.00661 eV, lower than their reference chromophore (0.00736 eV) except for **TPD5**. These findings indicate that **TPD1**–**TPD6** excluding **TPD5** possess excellent electron transport capability. The *λe* decreasing order for investigated compounds were found to be **TPD5 > TPDR > TPD6 > TPD3 > TPD2 > TPD1 > TPD4**.

Similarly, Table 4 shows the calculated reorganization energy of hole (*λ*_*h*_) for **TPDR** and **TPD1**–**TPD6** is found to be 0.00342, 0.00681, 0.00697, 0.00701, 0.00679, 0.00789 and 0.00785 eV, respectively. All the derivatives exhibited higher value of *λ*_*h*_ than **TPDR**, indicating the lower hole transport capability between D and A. The order of *λ*_*h*_ is **TPD5 > TPD6 > TPD3 > TPD2 > TPD1 > TPD4 > TPDR**. Overall, investigations reveal that the λ_*e*_ results of all the entitled molecules except for the reference are smaller than λ_h_ results which specifies that all acceptors are fascinating candidates for the transfer of electrons.

### Open-circuit voltage (*Voc*)

To analyze the maximum working capacity of OSCs, open-circuit voltage (*Voc*) plays a vital role. It is the determination of entire quantity of current that is generated by an optical material^47^. A higher value of *Voc* can be attained whereas the LUMO level of the acceptor has a higher energy value and the HOMO of the donor has a lower value^48^. By utilizing following Equation, *Voc* can be calculated^46^.

1 $$Voc = \left( {\left| {{\text{E}}_{{{\text{HOMO}}}}^{{\text{D}}} } \right| - \left| {{\text{E}}_{{{\text{LUMO}}}}^{{\text{A}}} } \right|} \right) - 0.3$$

**Figure 7:**
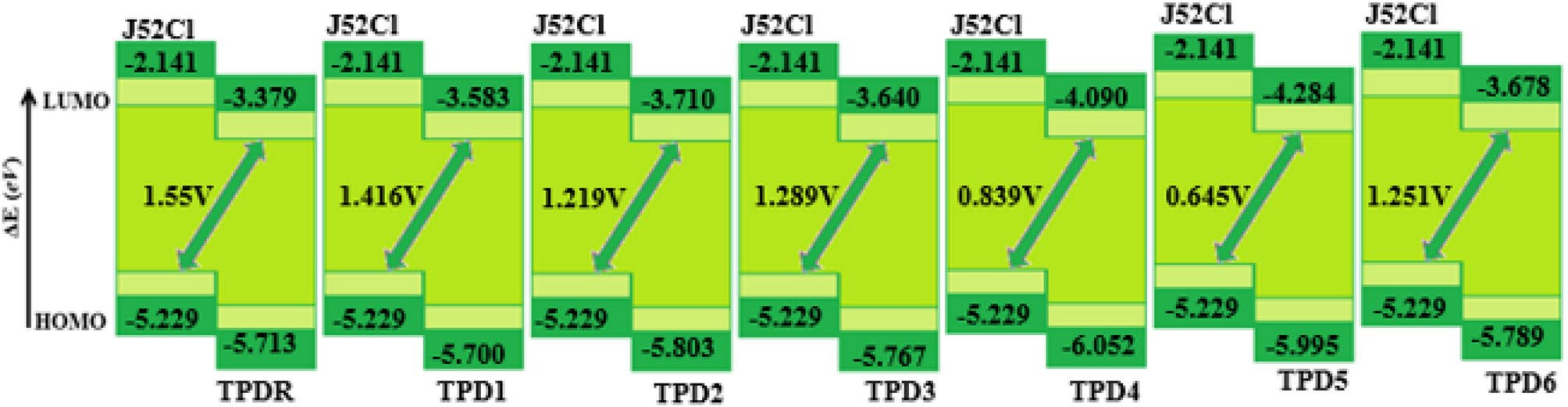
Graphical representation of *Voc* for entitled chromophores with **J52Cl**. All out put files of entitled compounds were accomplished by Gaussian 09 version D.01 (https://gaussian.com/g09citation/).

In this study, the chief purpose of Voc is to arrange the HOMO of well-known donor compound **J52Cl** with the LUMO of the acceptor^30^. The outcomes achieved from Eq. (1) are presented in Table 5 and Fig. 7.

**Table 5:** Open circuit voltage and energy driving force of the entitled compounds.

Compounds	*V*_*OC*_ (V)
**TPDR**	1.55
**TPD1**	1.42
**TPD2**	1.22
**TPD3**	1.29
**TPD4**	0.84
**TPD5**	0.64
**TPD6**	1.25

Table 5 reveals that **TPD1**, **TPD2, TPD3, and TPD6** have comparable values of *V*_*OC*_ in the range of 1.22–1.42 V with the reference chromophore TDPR (1.55 V), while **TPD4** and **TPD5** have value less than reference chromophore. Highest results of voltage is calculated in **TPD1** among all our compounds, may be due effective withdrawing groups with perfect planner geometry that facilitated the supreme shifting of chargers from D to A. *V*_*OC*_ order of entitled chromophores is found to be **TPDR** > **TPD1** > **TPD3 > TPD6 > TPD2 > TPD4 > TPD5**. A significant value of voltage is obtained for these chromophores which illustrated them as beneficial candidates for NF-OSCs.

### Charge transfer analysis

**Figure 8:**
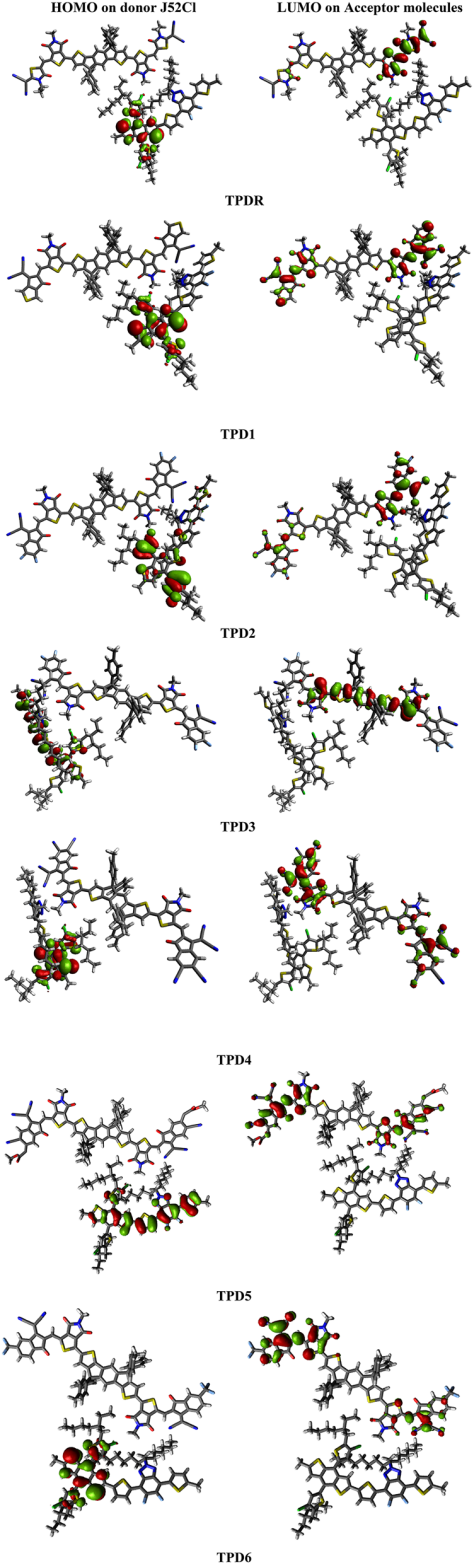
Charge transfer between ${\text{HOMO}}_{{{\mathbf{J}}52{\mathbf{Cl}}}}$ and LUMO of investigated molecule (**TPDR** and **TPD1**–**TPD6**) drawn with the help of Avogadro software, Version 1.2.0. (http://avogadro.cc/). All out put files of entitled compounds were accomplished by Gaussian 09 version D.01 (https://gaussian.com/g09citation/).

To predict the potential usage of designed compounds with regards to charge transfer characteristics for OSCs, the studied molecules **TPDR** and **TPD1**–**TPD6** are blended with **J52Cl** polymer and complex is optimized using above mentioned level of theory. In complex [**J52Cl**: **TPD1**–**TPD6 ]**, designed molecules are used as acceptor materials while **J52Cl** is used as donor material which is recognized as a well-known polymeric natured compound and frequently utilized in the CT analysis^30^. The effective charge density for HOMO is located at donor polymer **J52Cl**, whereas LUMO is concentrated over the terminal acceptor unit of **TPDR** and **TPD1**–**TPD6** as displayed in Fig. 8. The transfer of electronic charge rom D to A provides strong indication of charge mobility from D to A moiety. This charge transfers from D to A provides a piece of information that all our designed derivatives may be used as an efficient acceptor compounds for OSC.

### Transition density matrix and exciton binding energy

**Figure 9:**
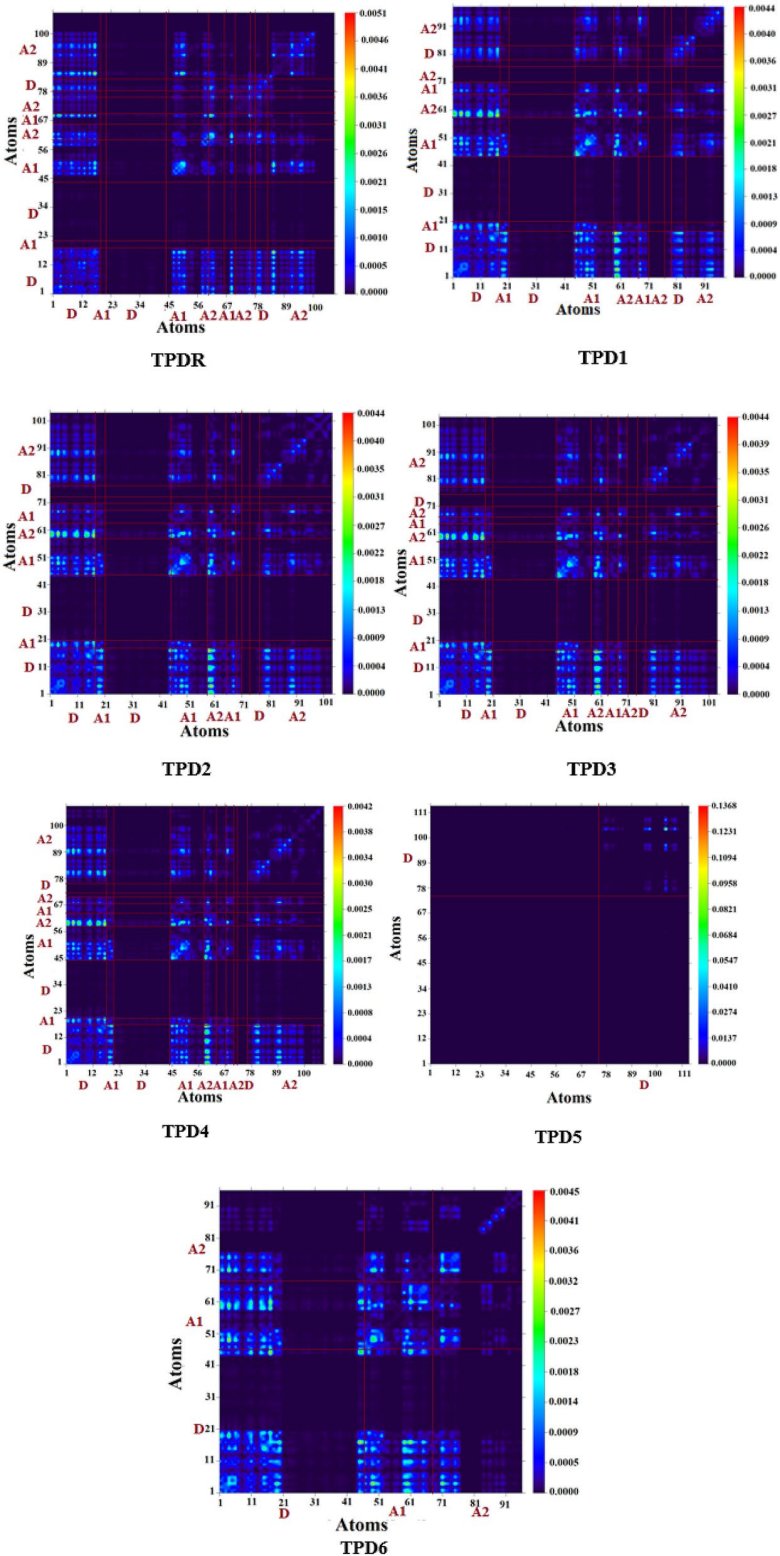
TDM of studied compounds at the S1 state. These were drawn with the help of Multiwfn 3.7software (http://sobereva.com/multiwfn/). All out put files of designed compounds were accomplished by Gaussian 09 version D.01 (https://gaussian.com/g09citation/).

The interpretation of transition processes in entitled chromophores may also be evaluated by calculating transition density matrix **(**TDM). The MPW1PW91/6-31G(d,p) level of theory was employed to estimate the behavior of transitions, essentially from the ground state (S_0_) to an excited state (S_1_) and interaction between donor and acceptor unit along with electron–hole localization. The hydrogen atoms effect is ignored because of its minute influence in these transitions. For TDM analysis, we divided our molecules (**TPDR** and **TPD1**–**TPD6**) into three fragments namely; central core donor (D), acceptor-1 (A_1_) and terminal acceptor-2 (A_2_) and their pictographs are shown in Fig. 9.

These TDM heat maps illustrated an efficient diagonal charge transfer coherence in all the designed chromophores. Electron coherence successfully transferred from D to A_1_ which facilitated the shifting of electron density towards A_2_ without trapping. Interestingly, in TDM map of **TPD5**, charge is observed with unique pattern only at the D part and this behavior is completely different than **TPDR** and **TPD1**, **TPD2, TPD3, TPD4** and **TPD6.** This unique pattern may generate interesting debate as to the origin of this pattern amongst readers of this journal. Nevertheless, findings of TDM heat maps excluding **TPD5** implies an facile, easier and higher exciton dissociation in the excited state which would help future solar cell development.

Binding energy (E_b_) is also considered a vital factor for evaluating the photovoltaic properties of OSCs particularly exciton dissociation capacity^49,50^. Binding energy is a noticeable parameter for determining the columbic force interaction among hole and electrons. In the excited state, lower columbic interaction between electron and hole and greater the exciton dissociation^51,52^. *E*_*opt*_ is a term that refers to the energy of the S_0_–S_1_^51,52^. Equation (2) is used to measure *E*_*b*_.

2 $$E_{b} = \, E_{H - L} E_{opt}$$

All designed molecules in comparison to **TPDR** have smaller E_b_ except **TPD5** (Table 6). This lowering E_b_ illustrates higher exciton dissociation in the excited state. The descending order of binding energy of all molecular is reported as **TPD5** > **TPDR** > **TPD6** > **TPD3** = **TPD2** > **TPD1** > **TPD4**. Among all the designed chromophores, **TPD3** and **TPD2** manifest lower binding energy that describes their highest efficiency in exciton dissociation with better optoelectronic properties. The chromophores with 1.9 eV E_b_ could be perfect as an OSCs material with significant *Voc.*

**Table 6:** Calculated $E_{H - L}$*,* $E_{opt}$, and $E_{b}$ of **TPDR** and **TPD1**–**TPD6**.

Compounds	E_H–L_ (eV)	E_opt_ (eV)	E_b_ (eV)
**TPDR**	2.33	1.94	0.39
**TPD1**	2.12	1.76	0.35
**TPD2**	2.09	1.73	0.36
**TPD3**	2.13	1.77	0.36
**TPD4**	1.96	1.63	0.33
**TPD5**	1.71	0.89	0.82
**TPD6**	2.11	1.74	0.37

## Conclusion

The solar light active organic chromophores (**TPD1**–**TPD6)** are modelled on the compound (**TPDR**) using efficient end-capped electron accepting groups. Fortunately, photovoltaic properties of the designed compounds show significant improvement on the parent compound. The designed molecules exhibit lower band gap in the range of 2.12–1.71 eV*,* while **TPDR** is found with 2.33 eV band gap. Moreover, **TPD1**–**TPD6** are found with broader absorption spectrum as compared to the reference molecule. Interestingly, **TPD5** yielded least ΔE value as 1.71 eV*,* where it is found 0.62 eV less than **TPDR** among designed compounds owing to the high electron withdrawing influence of end-capped acceptor cyano and ester groups with extended conjugation. Further, the *Voc* values are also estimated with regards to ${\text{HOMO}}_{{{\text{J}}52{\text{Cl}}}} - {\text{LUMO}}_{{{\text{Acceptor}}}}$ showing the order; **TPDR** (1.55 eV) > **TPD1** (1.42 eV) > **TPD3** (1.29 eV) > **TPD6** (1.25 eV) > **TPD2** (1.22 eV) > **TPD4** (0.84 eV) > **TPD5** (0.64 eV). Interestingly, $\lambda_{{\text{e}}}$ value of all the entitled chromophores is examined to be lower than $\lambda_{{\text{h}}}$ except for the reference compound which indicates the higher electron mobility rate in these compounds. Further, lower binding energy (0.33–0.82 eV) of studied molecules are obtained which define higher excitation dissociation. Subsequently, the higher excitation dissociation factor predicting the higher power conversion efficiency of entitled compounds. It is concluded that entitled chromophores obtained by modeling route could be appealing as efficient economically viable organic solar cell materials.

## Methods

The Gaussian 09 package was used for the analysis^53^ and the GaussView 5.0^54^ was employed for DFT calculations. The diverse DFT based levels of theory were applied such as B3LYP^55^, CAM-B3LYP^56^, MPW1PW91^57^ and $\omega$B97XD^58^ with 6-31G(d,p) basis set combinations for the optimisation of **TPDR** chromophores. The optimized geometries of **TPDR** were further used for UV–Vis analysis via TDDFT calculations at aforesaid levels and basis set in chloroform. Subsequently, MPW1PW91 level of theory exhibited absorption maximum values in good agreement with obtained experimental values (see Fig. 1). Thus, MPW1PW91 level was selected for further computations of **TPD1**–**TPD6** chromophores.

To study structure activity relationship and to explore the optoelectronic properties of entitled chromophores, the density of states (DOS), absorption spectra, FMOs analysis, reorganization energy (RE), open-circuit voltage (*Voc*) and transition density matrices (TDM) were investigated. However, charge transfer analysis was executed using aforesaid level of theory with a 3-21G basis set due to the larger size of complexes (**J52CL**: **TPD1**–**TPD6**). The reorganization energy has two major categories: external reorganization energy (*λ*_*ext*_*.*) which is used to explain the exterior environmental change and internal reorganization energy (*λ*_*int*_*.*) agreements with the internal structure rapid variations. In our study, external environmental influence is ignored as it has not much effect. Therefore, following Eq. (3) is used for the calculating the the reorganization energy of electron (*λ*_*e*_).

3 $$\lambda_{e} = \left[ {E_{0}^{ - } - E_{ - } } \right] + \left[ {E_{ - }^{0} - E_{0} } \right]$$

where $E_{ - }^{0}$ is neutral molecule energy obtained via anionic optimized structure, $E_{ - }^{ }$ is the energy of anions, $E_{0}^{ - }$ is the single point energy (SPE) of anions and $E_{0}^{ }$ is the SPE of neutral molecule. In the same way, reorganization energy of the hole (*λ*_*h*_) can be computed using Eq. (4)^59–61^.

4 $$\lambda_{h} = \left[ {E_{0}^{ + } - E_{ + } } \right] + \left[ {E_{ + }^{0} - E_{0} } \right]$$

Here $E_{ + }^{0}$ is the neutral molecule energy obtained via optimized cationic structure, $E_{ + }^{ }$ is the energy of cations and $E_{0}^{ + }$ is the SPE of cations^47^. Various software’s including GaussView^54^, PyMOlyze^62^, Multiwfn 3.7^63^, Avogadro^64^, and Chemcraft^65^ were used for data analyses.

## Supplementary Information

Supplementary Information.

## References

[1] Irfan M, Eliason B, Mahr MS, Iqbal J (2018). Tuning the optoelectronic properties of naphtho-dithiophene-based A–D–A type small donor molecules for bulk hetero-junction organic solar cells. ChemistrySelect.

[2] Burton T, Jenkins N, Sharpe D, Bossanyi E (2011). Wind Energy Handbook.

[3] Paish O (2002). Small hydro power: Technology and current status. Renew. Sustain. Energy Rev..

[4] Mahmood A, Yang J, Hu J, Wang X, Tang A, Geng Y, Zeng Q, Zhou E (2018). Introducing four 1, 1-dicyanomethylene-3-indanone end-capped groups as an alternative strategy for the design of small-molecular nonfullerene acceptors. J. Phys. Chem. C.

[5] Mahmood A, Khan SU-D, Rana UA (2014). Theoretical designing of novel heterocyclic azo dyes for dye sensitized solar cells. J. Comput. Electron..

[6] Pillai S, Catchpole K, Trupke T, Green M (2007). Surface plasmon enhanced silicon solar cells. J. Appl. Phys..

[7] Mehboob MY, Khan MU, Hussain R, Ayub K, Sattar A, Ahmad MK, Irshad Z, Adnan M (2021). Designing of benzodithiophene core-based small molecular acceptors for efficient non-fullerene organic solar cells. Spectrochim. Acta. A Mol. Biomol. Spectrosc..

[8] Yu G, Gao J, Hummelen JC, Wudl F, Heeger AJ (1995). Polymer photovoltaic cells: Enhanced efficiencies via a network of internal donor–acceptor heterojunctions. Science.

[9] Liu T, Troisi A (2013). What makes fullerene acceptors special as electron acceptors in organic solar cells and how to replace them. Adv. Mater..

[10] Holliday S, Ashraf RS, Nielsen CB, Kirkus M, Röhr JA, Tan C-H, Collado-Fregoso E, Knall A-C, Durrant JR, Nelson J (2015). A rhodanine flanked nonfullerene acceptor for solution-processed organic photovoltaics. J. Am. Chem. Soc..

[11] Sivula K, Luscombe CK, Thompson BC, Fréchet JM (2006). Enhancing the thermal stability of polythiophene: Fullerene solar cells by decreasing effective polymer regioregularity. J. Am. Chem. Soc..

[12] Zhang Y, Yip H-L, Acton O, Hau SK, Huang F, Jen AK-Y (2009). A simple and effective way of achieving highly efficient and thermally stable bulk-heterojunction polymer solar cells using amorphous fullerene derivatives as electron acceptor. Chem. Mater..

[13] Lin Y, Zhan X (2016). Oligomer molecules for efficient organic photovoltaics. Acc. Chem. Res..

[14] Adhikari T, Rahami ZG, Nunzi J-M, Lebel OJOE (2016). Synthesis, characterization and photovoltaic performance of novel glass-forming perylenediimide derivatives. Org. Electron..

[15] Adhikari T, Nunzi J-M, Lebel OJOE (2017). Solid-state showdown: Comparing the photovoltaic performance of amorphous and crystalline small-molecule diketopyrrolopyrrole acceptors. Org. Electron..

[16] Adhikari T, Nunzi J-M, Lebel OJOE (2017). Towards amorphous solution-processed small-molecule photovoltaic cells by design. Org. Electron..

[17] Adhikari T, Shahiduzzaman M, Yamamoto K, Lebel O, Nunzi J-MJSEM, Cells S (2017). Interfacial modification of the electron collecting layer of low-temperature solution-processed organometallic halide photovoltaic cells using an amorphous perylenediimide. Sol. Energy Mater. Sol. Cells.

[18] Sicot L, Fiorini C, Lorin A, Raimond P, Sentein C, Nunzi J-M (2000). Improvement of the photovoltaic properties of polythiophene-based cells. Sol. Energy Mater. Sol. Cells.

[19] Li G, Li D, Ma R, Liu T, Luo Z, Cui G, Tong L, Zhang M, Wang Z, Liu F (2020). Efficient modulation of end groups for the asymmetric small molecule acceptors enabling organic solar cells with over 15% efficiency. J. Mater. Chem. A.

[20] Hou J, Inganäs O, Friend RH, Gao F (2018). Organic solar cells based on non-fullerene acceptors. Nat. Mater..

[21] Cheng P, Li G, Zhan X, Yang Y (2018). Next-generation organic photovoltaics based on non-fullerene acceptors. Nat. Photonics.

[22] Bibi S, Li P, Zhang J (2013). X-shaped donor molecules based on benzo [2, 1-b: 3, 4-b′] dithiophene as organic solar cell materials with PDIs as acceptors. J. Mater. Chem. A.

[23] Ripaud E, Rousseau T, Leriche P, Roncali J (2011). Unsymmetrical triphenylamine-oligothiophene hybrid conjugated systems as donor materials for high-voltage solution-processed organic solar cells. Adv. Energy Mater..

[24] Takacs CJ, Sun Y, Welch GC, Perez LA, Liu X, Wen W, Bazan GC, Heeger AJ (2012). Solar cell efficiency, self-assembly, and dipole–dipole interactions of isomorphic narrow-band-gap molecules. J. Am. Chem. Soc..

[25] Li M, Liu Y, Ni W, Liu F, Feng H, Zhang Y, Liu T, Zhang H, Wan X, Kan B (2016). A simple small molecule as an acceptor for fullerene-free organic solar cells with efficiency near 8%. J. Mater. Chem. A.

[26] Qiu N, Zhang H, Wan X, Li C, Ke X, Feng H, Kan B, Zhang H, Zhang Q, Lu Y (2017). A new nonfullerene electron acceptor with a ladder type backbone for high-performance organic solar cells. Adv. Mater..

[27] Mao J, He N, Ning Z, Zhang Q, Guo F, Chen L, Wu W, Hua J, Tian H (2012). Stable dyes containing double acceptors without COOH as anchors for highly efficient dye-sensitized solar cells. Angew. Chem..

[28] Wu Y, Zhang X, Li W, Wang ZS, Tian H, Zhu W (2012). Hexylthiophene-featured D–A–π–A structural indoline chromophores for coadsorbent-free and panchromatic dye-sensitized solar cells. Adv. Energy Mater..

[29] Thomas KJ, Singh P, Baheti A, Hsu Y-C, Ho K-C, Lin JTS (2011). Electro-optical properties of new anthracene based organic dyes for dye-sensitized solar cells. Dyes Pigments.

[30] Li J, Li F, Zhang B, Zhou E (2020). Synthesis of 1-formyl-3-bromo-thieno [3, 4-c] pyrrole-4, 6-dione and the application in A2–A1–D–A1–A2 type non-fullerene acceptor. J. Phys. Chem. C.

[31] Khan MU, Khalid M, Ibrahim M, Braga AAC, Safdar M, Al-Saadi AA, Janjua MRSA (2018). First theoretical framework of triphenylamine–dicyanovinylene-based nonlinear optical dyes: Structural modification of π-linkers. J. Phys. Chem. C.

[32] Janjua MRSA, Khan MU, Bashir B, Iqbal MA, Song Y, Naqvi SAR, Khan ZA (2012). Effect of π-conjugation spacer (C C) on the first hyperpolarizabilities of polymeric chain containing polyoxometalate cluster as a side-chain pendant: A DFT study. Comput. Theor. Chem..

[33] Janjua MRSA, Amin M, Ali M, Bashir B, Khan MU, Iqbal MA, Guan W, Yan L, Su ZM (2012). A DFT study on the two-dimensional second-order nonlinear optical (NLO) response of terpyridine-substituted hexamolybdates: Physical insight on 2D inorganic–organic hybrid functional materials. Eur. J. Inorg. Chem..

[34] Khan MU, Ibrahim M, Khalid M, Qureshi MS, Gulzar T, Zia KM, Al-Saadi AA, Janjua MRSA (2019). First theoretical probe for efficient enhancement of nonlinear optical properties of quinacridone based compounds through various modifications. Chem. Phys. Lett..

[35] Khan MU, Ibrahim M, Khalid M, Braga AAC, Ahmed S, Sultan A (2019). Prediction of second-order nonlinear optical properties of D–p–A compounds containing novel fluorene derivatives: A promising route to giant hyperpolarizabilities. J. Cluster Sci..

[36] Khan MU, Ibrahim M, Khalid M, Jamil S, Al-Saadi AA, Janjua MRSA (2019). Quantum chemical designing of indolo [3, 2, 1-jk] carbazole-based dyes for highly efficient nonlinear optical properties. Chem. Phys. Lett..

[37] Janjua MRSA, Khan MU, Khalid M, Ullah N, Kalgaonkar R, Alnoaimi K, Baqader N, Jamil S (2020). Theoretical and conceptual framework to design efficient dye-sensitized solar cells (DSSCs): Molecular engineering by DFT method. J. Cluster Sci..

[38] Khan MU, Hussain R, Mehboob MY, Khalid M, Ehsan MA, Rehman A, Janjua MRSA (2021). First theoretical framework of Z-shaped acceptor materials with fused-chrysene core for high performance organic solar cells. Spectrochim. Acta. A Mol. Biomol. Spectrosc..

[39] Mahmood A, Khan SUD, Rana UA, Tahir MH (2014). Red shifting of absorption maxima of phenothiazine based dyes by incorporating electron-deficient thiadiazole derivatives as π-spacer. Arab. J. Chem..

[40] Mahmood A, Khan SUD, Rana UA, Janjua MRSA, Tahir MH, Nazar MF, Song Y (2015). Effect of thiophene rings on UV/visible spectra and non-linear optical (NLO) properties of triphenylamine based dyes: A quantum chemical perspective. J. Phys. Org. Chem..

[41] Mahmood A (2019). Photovoltaic and charge transport behavior of diketopyrrolopyrrole based compounds with A-D–A–D–A skeleton. J. Cluster Sci..

[42] Khan MU, Mehboob MY, Hussain R, Afzal Z, Khalid M, Adnan M (2020). Designing spirobifullerene core based three-dimensional cross shape acceptor materials with promising photovoltaic properties for high-efficiency organic solar cells. Int. J. Quantum Chem..

[43] Khan MU, Mehboob MY, Hussain R, Fatima R, Tahir MS, Khalid M, Braga AAC (2021). Molecular designing of high-performance 3D star-shaped electron acceptors containing a truxene core for nonfullerene organic solar cells. J. Phys. Org. Chem..

[44] Yao C, Yang Y, Li L, Bo M, Zhang J, Peng C, Huang Z, Wang J (2020). Elucidating the key role of the cyano (−C≡N) group to construct environmentally friendly fused-ring electron acceptors. J. Phys. Chem. C.

[45] Mahmood A, Abdullah MI, Khan SU-D (2015). Enhancement of nonlinear optical (NLO) properties of indigo through modification of auxiliary donor, donor and acceptor. Spectrochim. Acta. A Mol. Biomol. Spectrosc..

[46] Scharber MC, Mühlbacher D, Koppe M, Denk P, Waldauf C, Heeger AJ, Brabec CJ (2006). Design rules for donors in bulk-heterojunction solar cells—Towards 10% energy-conversion efficiency. Adv. Mater..

[47] Tang S, Zhang J (2012). Design of donors with broad absorption regions and suitable frontier molecular orbitals to match typical acceptors via substitution on oligo (thienylenevinylene) toward solar cells. J. Comput. Chem..

[48] Bai H, Wang Y, Cheng P, Li Y, Zhu D, Zhan X (2014). Acceptor–donor–acceptor small molecules based on indacenodithiophene for efficient organic solar cells. ACS Appl. Mater. Interfaces.

[49] Kraner S, Prampolini G, Cuniberti G (2017). Exciton binding energy in molecular triads. J. Phys. Chem. C.

[50] Kraner S, Scholz R, Plasser F, Koerner C, Leo K (2015). Exciton size and binding energy limitations in one-dimensional organic materials. J. Chem. Phys..

[51] Köse ME (2012). Evaluation of acceptor strength in thiophene coupled donor–acceptor chromophores for optimal design of organic photovoltaic materials. J. Phys. Chem. A.

[52] Dkhissi A (2011). Excitons in organic semiconductors. Synth. Met..

[53] Frisch MJ, Trucks GW, Schlegel HB, Scuseria G, Robb MA, Cheeseman JR, Scalmani G, Barone V, Mennucci B, Petersson G, Nakatsuji H, Caricato M, Li X, Hratchian HP, Izmaylov AF, Bloino J, Zheng G, Sonnenberg JL, Hada M, Ehara M, Toyota K, Fukuda R, Hasegawa J, Ishida M, Nakajima T, Honda Y, Kitao O, Nakai H, Vreven T, Montgomery JA, Peralta JE, Ogliaro F, Bearpark M, Heyd JJ, Brothers E, Kudin KN, Staroverov VN, Kobayashi R, Normand J, Raghavachari K, Rendell A, Burant JC, Iyengar SS, Tomasi J, Cossi M, Rega N, Millam JM, Klene M, Knox JE, Cross JB, Bakken V, Adamo C, Jaramillo J, Gomperts R, Stratmann RE, Yazyev O, Austin AJ, Cammi R, Pomelli C, Ochterski JW, Martin RL, Morokuma K, Zakrzewski VJ, Voth GA, Salvador P, Dannenberg JJ, Dapprich S, Daniels AD, Farkas O, Foresman JB, Ortiz JV, Cioslowski J, Fox DJ (2009). D. 0109, Revision D. 01.

[54] Dennington RD, Keith TA, Millam JM (2008). GaussView 5.0. 8.

[55] Civalleri B, Zicovich-Wilson CM, Valenzano L, Ugliengo P (2008). B3LYP augmented with an empirical dispersion term (B3LYP-D*) as applied to molecular crystals. CrystEngComm.

[56] Yanai T, Tew DP, Handy NC (2004). A new hybrid exchange–correlation functional using the Coulomb-attenuating method (CAM-B3LYP). Chem. Phys. Lett..

[57] Adamo C, Barone V (1998). Exchange functionals with improved long-range behavior and adiabatic connection methods without adjustable parameters: The m PW and m PW1PW models. J. Chem. Phys..

[58] Chai J-D, Head-Gordon M (2008). Long-range corrected hybrid density functionals with damped atom–atom dispersion corrections. Phys. Chem. Chem. Phys..

[59] Khan MU, Iqbal J, Khalid M, Hussain R, Braga AAC, Hussain M, Muhammad S (2019). Designing triazatruxene-based donor materials with promising photovoltaic parameters for organic solar cells. RSC Adv..

[60] Khan MU, Khalid M, Arshad MN, Khan MN, Usman M, Ali A, Saifullah B (2020). Designing star-shaped subphthalocyanine-based acceptor materials with promising photovoltaic parameters for non-fullerene solar cells. ACS Omega.

[61] Khan MU, Hussain R, Yasir Mehboob M, Khalid M, Shafiq Z, Aslam M, Al-Saadi AA, Jamil S, Janjua MRSA (2020). In silico modeling of new “Y-Series”-based near-infrared sensitive non-fullerene acceptors for efficient organic solar cells. ACS Omega.

[62] O’boyle NM, Tenderholt AL, Langner KM (2008). Cclib: A library for package-independent computational chemistry algorithms. J. Comput. Chem..

[63] Lu T, Chen F (2012). Multiwfn: A multifunctional wavefunction analyzer. J. Comput. Chem..

[64] Hanwell MD, Curtis DE, Lonie DC, Vandermeersch T, Zurek E, Hutchison GR (2012). Avogadro: An advanced semantic chemical editor, visualization, and analysis platform. J. Cheminform..

[65] Zhurko, G. A. & D. A. Zhurko. ChemCraft, version 1.6. URL: http://www.chemcraftprog.com (2009).

