# Supplementary material for: Exploration of efficient electron acceptors for organic solar cells: rational design of indacenodithiophene based non-fullerene compounds
Source: Sci Rep. 2021 Oct 7;11:19931. doi: 10.1038/s41598-021-99254-4 (PMC8497501; doi:10.1038/s41598-021-99254-4)
Supplement: Supplementary file 1 — Supplementary Information. [file 41598_2021_99254_MOESM1_ESM.docx]

# Supplementary Information

**Exploration of Efficient Electron Acceptors for Organic Solar Cells: Rational Designing of Indacenodithiophene based Non-Fullerene Compounds**

Muhammad Khalid,*^1^ Muhammad Usman Khan,*^2^ Eisha-tul-Razia,^1^ Zahid Shafiq,^3^ Mohammed Mujahid Alam,^4^ Muhammad Imran,^4^ Muhammad Safwan Akram*^5^

*^1^Department of Chemistry, Khwaja Fareed University of Engineering & Information Technology, Rahim Yar Khan, 64200, Pakistan*

*^2^Department of Chemistry, University of Okara, Okara-56300, Pakistan*

^3^Institute of Chemical Sciences, Bahauddin Zakariya University, Multan, 60800, Pakistan

*^4^Department of Chemistry, Faculty of Science, King Khalid University, P.O. Box 9004, Abha 61413, Saudi Arabia.*

^5^School of Health and Life Sciences, Teesside University, Middlesbrough, TS1 3BA, UK

^6^National Horizons Centre, Teesside University, Darlington, DL1 1HG, UK

*Corresponding authors E-mail addresses:

Dr. Muhammad Khalid ([muhammad.khalid@kfueit.edu.pk](mailto:muhammad.khalid@kfueit.edu.pk); [Khalid@iq.usp.br](mailto:Khalid@iq.usp.br))

Dr. Muhammad Safwan Akram (Safwan.akram@tees.ac.uk)

Dr. Muhammad Usman Khan ([usman.chemistry@gmail.com](mailto:usman.chemistry@gmail.com) ; usmankhan@uo.edu.pk)

**Table S1:** Cartesian coordinates of **TPDR**

| **Atom** | **X-axis** | **Y-axis** | **Z-axis** |
| --- | --- | --- | --- |
| C | -2.69349 | -0.7598 | 0.00424 |
| C | -3.38643 | 0.434841 | -0.02221 |
| C | -4.77312 | 0.253709 | -0.0298 |
| C | -5.13378 | -1.08945 | -0.01712 |
| S | -3.71776 | -2.1316 | 0.014285 |
| C | -1.2698 | -0.53019 | 0.024701 |
| C | -1.08537 | 0.873179 | 0.030541 |
| C | -2.42999 | 1.616868 | 0.005421 |
| C | -0.18692 | -1.41412 | 0.020941 |
| C | 1.085374 | -0.87322 | 0.030211 |
| C | 1.269804 | 0.53015 | 0.02472 |
| C | 0.186918 | 1.414079 | 0.021291 |
| C | 2.42999 | -1.6169 | 0.004759 |
| C | 3.386427 | -0.43487 | -0.02263 |
| C | 2.693486 | 0.759766 | 0.004195 |
| C | 4.773118 | -0.25373 | -0.03033 |
| C | 5.133774 | 1.089425 | -0.01735 |
| S | 3.717754 | 2.131565 | 0.014459 |
| C | -6.46779 | -1.61031 | -0.02642 |
| C | 6.467782 | 1.610285 | -0.02659 |
| C | -2.48584 | 2.476438 | -1.26349 |
| C | -2.70536 | 2.429291 | 1.277521 |
| C | 2.705478 | -2.42967 | 1.276608 |
| C | 2.485716 | -2.47612 | -1.26439 |
| C | 3.769861 | -3.33802 | 1.298404 |
| C | 4.096217 | -4.02908 | 2.456903 |
| C | 3.374472 | -3.84267 | 3.639551 |
| C | 2.319566 | -2.93153 | 3.615422 |
| C | 1.988955 | -2.23474 | 2.456377 |
| C | 1.976877 | -3.77782 | -1.26193 |
| C | 1.942488 | -4.53284 | -2.42809 |
| C | 2.416339 | -4.02063 | -3.63767 |
| C | 2.919142 | -2.71861 | -3.63607 |
| C | 2.952942 | -1.95768 | -2.47283 |
| C | -3.76971 | 3.337669 | 1.299651 |
| C | -4.09594 | 4.02843 | 2.458364 |
| C | -3.37411 | 3.841666 | 3.640904 |
| C | -2.31924 | 2.930481 | 3.616443 |
| C | -1.98875 | 2.234006 | 2.45718 |
| C | -1.97691 | 3.778098 | -1.26074 |
| C | -1.94264 | 4.533436 | -2.42669 |
| C | -2.4167 | 4.021598 | -3.63635 |
| C | -2.9196 | 2.719611 | -3.63504 |
| C | -2.95328 | 1.958368 | -2.47199 |
| S | 7.823846 | 0.494898 | -0.0498 |
| C | 9.017762 | 1.78377 | -0.05321 |
| C | 8.346079 | 2.98876 | -0.03568 |
| C | 6.948546 | 2.910092 | -0.02047 |
| S | -7.82384 | -0.49491 | -0.04995 |
| C | -9.01777 | -1.78378 | -0.05312 |
| C | -8.34609 | -2.98877 | -0.03538 |
| C | -6.94856 | -2.91011 | -0.02014 |
| C | 8.750241 | 4.412389 | -0.02925 |
| N | 7.549177 | 5.118755 | -0.01 |
| C | 6.417268 | 4.288735 | -0.0035 |
| C | -8.75027 | -4.41239 | -0.02867 |
| N | -7.54921 | -5.11877 | -0.00921 |
| C | -6.4173 | -4.28875 | -0.00289 |
| C | 10.44434 | 1.723745 | -0.07106 |
| C | -10.4443 | -1.72374 | -0.07102 |
| C | 11.32206 | 0.687429 | -0.08843 |
| C | 11.02352 | -0.74295 | -0.09048 |
| C | 13.36573 | -0.78008 | -0.1166 |
| S | 13.06123 | 0.945376 | -0.10713 |
| O | 9.922159 | -1.26011 | -0.07417 |
| C | 14.66977 | -1.23761 | -0.1261 |
| O | 5.278201 | 4.701035 | 0.012868 |
| O | 9.860697 | 4.902013 | -0.03832 |
| O | -9.86073 | -4.90201 | -0.03764 |
| O | -5.27823 | -4.70106 | 0.013563 |
| C | -11.3221 | -0.68742 | -0.08861 |
| C | -11.0235 | 0.742951 | -0.09094 |
| C | -13.3657 | 0.780104 | -0.11724 |
| S | -13.0612 | -0.94535 | -0.10731 |
| O | -9.92213 | 1.260106 | -0.07473 |
| C | -14.6697 | 1.237644 | -0.12696 |
| C | 7.466082 | 6.559816 | 0.002958 |
| C | -7.46613 | -6.55982 | 0.004057 |
| C | 3.710755 | -4.61819 | 4.882382 |
| C | 2.411208 | -4.85218 | -4.88981 |
| C | -3.71027 | 4.61686 | 4.883972 |
| C | -2.41167 | 4.853501 | -4.88825 |
| C | 15.71561 | -0.27257 | -0.13037 |
| N | 16.55469 | 0.532076 | -0.13347 |
| C | 15.06819 | -2.6001 | -0.12605 |
| N | 15.43819 | -3.70274 | -0.12216 |
| C | -15.7156 | 0.272618 | -0.13106 |
| N | -16.5547 | -0.53202 | -0.13403 |
| C | -15.0681 | 2.600137 | -0.12728 |
| N | -15.4381 | 3.702783 | -0.12367 |
| H | -5.49995 | 1.056448 | -0.04045 |
| H | -0.33996 | -2.48752 | 0.001627 |
| H | 0.339961 | 2.487484 | 0.002247 |
| H | 5.499944 | -1.05647 | -0.04131 |
| H | 4.34151 | -3.51597 | 0.393293 |
| H | 4.926863 | -4.72871 | 2.442264 |
| H | 1.741625 | -2.76033 | 4.518765 |
| H | 1.164009 | -1.53195 | 2.477941 |
| H | 1.614375 | -4.21219 | -0.33628 |
| H | 1.540842 | -5.54137 | -2.39541 |
| H | 3.290852 | -2.28855 | -4.56149 |
| H | 3.343253 | -0.94652 | -2.51032 |
| H | -4.34142 | 3.515887 | 0.394634 |
| H | -4.92656 | 4.728094 | 2.443983 |
| H | -1.74124 | 2.759018 | 4.519693 |
| H | -1.16383 | 1.531174 | 2.478483 |
| H | -1.61425 | 4.212189 | -0.33501 |
| H | -1.54091 | 5.541928 | -2.39379 |
| H | -3.29148 | 2.289837 | -4.56052 |
| H | -3.34368 | 0.947242 | -2.5097 |
| H | 10.8723 | 2.725406 | -0.07018 |
| H | -10.8723 | -2.7254 | -0.06995 |
| H | 8.48458 | 6.945546 | -0.004 |
| H | 6.927311 | 6.918321 | -0.87586 |
| H | 6.944998 | 6.90316 | 0.898325 |
| H | -8.48463 | -6.94555 | -0.00298 |
| H | -6.92721 | -6.91851 | -0.8746 |
| H | -6.9452 | -6.90299 | 0.899581 |
| H | 3.280748 | -5.62476 | 4.847072 |
| H | 4.791012 | -4.73233 | 5.001667 |
| H | 3.321619 | -4.12742 | 5.776821 |
| H | 1.594589 | -5.57753 | -4.88378 |
| H | 2.305739 | -4.23041 | -5.78167 |
| H | 3.345572 | -5.4142 | -4.99333 |
| H | -4.79052 | 4.73085 | 5.003465 |
| H | -3.32093 | 4.125926 | 5.778231 |
| H | -3.28038 | 5.623486 | 4.848822 |
| H | -1.59457 | 5.578317 | -4.88243 |
| H | -2.30708 | 4.231921 | -5.78035 |
| H | -3.34571 | 5.416176 | -4.99112 |
| N | -12.2063 | 1.495886 | -0.1192 |
| N | 12.2063 | -1.49588 | -0.11846 |
| C | -12.0906 | 2.958917 | -0.09701 |
| C | -12.1587 | 3.526445 | 1.311448 |
| H | -12.8644 | 3.375795 | -0.73899 |
| H | -11.1198 | 3.169254 | -0.547 |
| H | -12.0456 | 4.611673 | 1.267162 |
| H | -13.1166 | 3.312458 | 1.788064 |
| H | -11.3536 | 3.122529 | 1.928334 |
| C | 12.09063 | -2.9589 | -0.09591 |
| C | 12.15871 | -3.52608 | 1.312694 |
| H | 12.86448 | -3.37593 | -0.73774 |
| H | 11.11986 | -3.16936 | -0.5459 |
| H | 12.04557 | -4.61132 | 1.268672 |
| H | 13.11656 | -3.31196 | 1.789313 |
| H | 11.35355 | -3.12202 | 1.929433 |

**Table S2:** Cartesian coordinates of **TPD1**

| **Atom** | **X-axis** | **Y-axis** | **Z-axis** |
| --- | --- | --- | --- |
| C | -2.73568 | -0.58646 | 0.043864 |
| C | -3.3513 | 0.650891 | 0.019722 |
| C | -4.74553 | 0.558607 | 0.015016 |
| C | -5.19186 | -0.75987 | 0.027518 |
| S | -3.84421 | -1.89028 | 0.054212 |
| C | -1.30072 | -0.44725 | 0.06243 |
| C | -1.02731 | 0.941834 | 0.068644 |
| C | -2.32158 | 1.769792 | 0.045872 |
| C | -0.27704 | -1.39939 | 0.057944 |
| C | 1.02733 | -0.94184 | 0.068387 |
| C | 1.300746 | 0.447237 | 0.062484 |
| C | 0.277064 | 1.399379 | 0.058259 |
| C | 2.321598 | -1.7698 | 0.045356 |
| C | 3.351317 | -0.65089 | 0.019447 |
| C | 2.735705 | 0.586458 | 0.043892 |
| C | 4.745546 | -0.55861 | 0.014684 |
| C | 5.191881 | 0.75987 | 0.02744 |
| S | 3.844231 | 1.890276 | 0.054437 |
| C | -6.55641 | -1.19039 | 0.024189 |
| C | 6.556436 | 1.190384 | 0.024144 |
| C | -2.32546 | 2.630842 | -1.22319 |
| C | -2.5428 | 2.597361 | 1.318778 |
| C | 2.542869 | -2.59772 | 1.318025 |
| C | 2.325408 | -2.6305 | -1.22395 |
| C | 3.554378 | -3.56448 | 1.34469 |
| C | 3.836196 | -4.27207 | 2.504767 |
| C | 3.120274 | -4.04499 | 3.684032 |
| C | 2.117569 | -3.07703 | 3.654583 |
| C | 1.832086 | -2.36308 | 2.493892 |
| C | 1.744271 | -3.90136 | -1.22062 |
| C | 1.6652 | -4.65272 | -2.38718 |
| C | 2.164216 | -4.16728 | -3.59746 |
| C | 2.73906 | -2.89523 | -3.59678 |
| C | 2.818152 | -2.13825 | -2.43339 |
| C | -3.55431 | 3.564163 | 1.345717 |
| C | -3.83608 | 4.271448 | 2.505959 |
| C | -3.12014 | 4.044017 | 3.685183 |
| C | -2.11746 | 3.076085 | 3.655448 |
| C | -1.832 | 2.362416 | 2.494544 |
| C | -1.74421 | 3.901678 | -1.21954 |
| C | -1.66522 | 4.653378 | -2.38586 |
| C | -2.16443 | 4.168334 | -3.59625 |
| C | -2.73936 | 2.896343 | -3.59589 |
| C | -2.81837 | 2.139001 | -2.4327 |
| S | 7.835007 | -0.00728 | -0.00617 |
| C | 9.113264 | 1.203269 | 0.014473 |
| C | 8.514145 | 2.451869 | 0.023688 |
| C | 7.118391 | 2.460487 | 0.028101 |
| S | -7.83499 | 0.00727 | -0.00623 |
| C | -9.11324 | -1.20328 | 0.014515 |
| C | -8.51412 | -2.45188 | 0.023822 |
| C | -7.11837 | -2.46049 | 0.028219 |
| C | 9.00118 | 3.854344 | 0.005544 |
| N | 7.840742 | 4.630157 | 0.026162 |
| C | 6.666586 | 3.868857 | 0.036175 |
| C | -9.00115 | -3.85435 | 0.005837 |
| N | -7.84071 | -4.63016 | 0.026499 |
| C | -6.66656 | -3.86886 | 0.03641 |
| C | 10.52876 | 1.04796 | 0.072743 |
| C | -10.5287 | -1.04797 | 0.072756 |
| C | 11.33989 | -0.05253 | 0.00099 |
| C | 10.89038 | -1.44086 | -0.3034 |
| O | 9.740894 | -1.83994 | -0.36368 |
| O | 5.550319 | 4.340652 | 0.047546 |
| O | 10.13317 | 4.279996 | -0.03288 |
| O | -10.1331 | -4.28001 | -0.0325 |
| O | -5.55029 | -4.34065 | 0.047783 |
| C | -11.3399 | 0.05251 | 0.000847 |
| C | -10.8904 | 1.440809 | -0.30365 |
| O | -9.74087 | 1.839887 | -0.36393 |
| C | 7.845807 | 6.073788 | 0.020598 |
| C | -7.84577 | -6.07379 | 0.021079 |
| C | 3.408584 | -4.83644 | 4.928845 |
| C | 2.110984 | -4.99649 | -4.85002 |
| C | -3.40875 | 4.834989 | 4.930232 |
| C | -2.11126 | 4.998047 | -4.84847 |
| H | -5.41993 | 1.40591 | 0.006103 |
| H | -0.49961 | -2.46056 | 0.038269 |
| H | 0.499637 | 2.460551 | 0.038832 |
| H | 5.41995 | -1.40591 | 0.005531 |
| H | 4.119576 | -3.77413 | 0.442312 |
| H | 4.627068 | -5.0164 | 2.494367 |
| H | 1.545553 | -2.87376 | 4.555017 |
| H | 1.047465 | -1.61548 | 2.511601 |
| H | 1.360143 | -4.31513 | -0.29429 |
| H | 1.20798 | -5.63728 | -2.35401 |
| H | 3.132388 | -2.48617 | -4.52278 |
| H | 3.264975 | -1.15077 | -2.47141 |
| H | -4.11949 | 3.774075 | 0.44339 |
| H | -4.6269 | 5.015829 | 2.495753 |
| H | -1.5454 | 2.872597 | 4.555802 |
| H | -1.04736 | 1.614838 | 2.51204 |
| H | -1.35995 | 4.31513 | -0.29313 |
| H | -1.20791 | 5.637887 | -2.35244 |
| H | -3.13282 | 2.487602 | -4.52196 |
| H | -3.26528 | 1.151569 | -2.47097 |
| H | 11.01426 | 2.016382 | 0.173642 |
| H | -11.0142 | -2.01638 | 0.17376 |
| H | 8.886367 | 6.395392 | 0.027888 |
| H | 7.345821 | 6.454567 | -0.87183 |
| H | 7.33163 | 6.45819 | 0.902986 |
| H | -8.88632 | -6.39541 | 0.02859 |
| H | -7.34596 | -6.45467 | -0.87142 |
| H | -7.33141 | -6.45809 | 0.9034 |
| H | 2.937937 | -5.82432 | 4.885496 |
| H | 4.481715 | -4.9952 | 5.060968 |
| H | 3.029158 | -4.3318 | 5.819678 |
| H | 1.268362 | -5.69124 | -4.8334 |
| H | 2.01633 | -4.37006 | -5.73991 |
| H | 3.022673 | -5.59212 | -4.96692 |
| H | -4.48232 | 4.988155 | 5.065545 |
| H | -3.02394 | 4.333082 | 5.820289 |
| H | -2.94357 | 5.825345 | 4.884598 |
| H | -1.26545 | 5.688992 | -4.83399 |
| H | -2.02213 | 4.371759 | -5.73902 |
| H | -3.02056 | 5.597933 | -4.96219 |
| C | 12.11805 | -2.21724 | -0.53341 |
| C | 13.25341 | -1.38043 | -0.32306 |
| C | 12.4296 | -3.49564 | -0.88336 |
| H | 11.76903 | -4.32338 | -1.0941 |
| C | 14.43763 | -2.04096 | -0.51971 |
| H | 15.45214 | -1.68157 | -0.43754 |
| S | 14.13919 | -3.67849 | -0.96327 |
| C | 12.80594 | -0.04398 | 0.075169 |
| C | 13.64822 | 0.955861 | 0.498341 |
| C | 13.22501 | 2.208094 | 1.027321 |
| N | 12.9319 | 3.231927 | 1.492355 |
| C | 15.05948 | 0.75991 | 0.481411 |
| N | 16.20845 | 0.586397 | 0.45608 |
| C | -12.118 | 2.217177 | -0.53377 |
| C | -13.2534 | 1.380379 | -0.3234 |
| C | -14.4376 | 2.040893 | -0.52015 |
| H | -15.4521 | 1.68152 | -0.43799 |
| C | -12.4296 | 3.495543 | -0.88383 |
| H | -11.769 | 4.323268 | -1.09461 |
| S | -14.1391 | 3.678393 | -0.96382 |
| C | -12.8059 | 0.04396 | 0.074965 |
| C | -13.6482 | -0.95584 | 0.498184 |
| C | -13.225 | -2.20803 | 1.027287 |
| N | -12.932 | -3.23183 | 1.492427 |
| C | -15.0595 | -0.75989 | 0.481181 |
| N | -16.2085 | -0.58636 | 0.455797 |

**Table S3:** Cartesian coordinates of **TPD2**

| **Atom** | **X-axis** | **Y-axis** | **Z-axis** |
| --- | --- | --- | --- |
| C | 2.679819 | 0.797361 | 0.057387 |
| C | 3.388648 | -0.37163 | -0.14916 |
| C | 4.770696 | -0.16867 | -0.15664 |
| C | 5.113726 | 1.166554 | 0.038629 |
| S | 3.683832 | 2.170755 | 0.241901 |
| C | 1.261122 | 0.542678 | 0.077356 |
| C | 1.096317 | -0.85141 | -0.10726 |
| C | 2.449616 | -1.56212 | -0.26475 |
| C | 0.167318 | 1.400718 | 0.225793 |
| C | -1.09694 | 0.842564 | 0.196233 |
| C | -1.26197 | -0.5498 | -0.00047 |
| C | -0.16854 | -1.40651 | -0.15862 |
| C | -2.45139 | 1.557686 | 0.321775 |
| C | -3.39144 | 0.373276 | 0.160577 |
| C | -2.68136 | -0.80123 | -0.00623 |
| C | -4.77322 | 0.1689 | 0.176888 |
| C | -5.11521 | -1.17074 | 0.012866 |
| S | -3.68413 | -2.18031 | -0.15132 |
| C | 6.439791 | 1.700905 | 0.076913 |
| C | -6.44143 | -1.70481 | -0.02335 |
| C | 2.489794 | -2.23784 | -1.64082 |
| C | 2.765665 | -2.53433 | 0.879493 |
| C | -2.69037 | 2.175935 | 1.705815 |
| C | -2.56862 | 2.586973 | -0.80888 |
| C | -3.76247 | 3.05483 | 1.894665 |
| C | -4.05523 | 3.564695 | 3.152113 |
| C | -3.29032 | 3.220074 | 4.270232 |
| C | -2.22701 | 2.338862 | 4.078417 |
| C | -1.93033 | 1.823293 | 2.82003 |
| C | -2.06308 | 3.879849 | -0.64417 |
| C | -2.0876 | 4.793915 | -1.69022 |
| C | -2.61984 | 4.45545 | -2.93647 |
| C | -3.1195 | 3.16245 | -3.0971 |
| C | -3.09414 | 2.242511 | -2.05464 |
| C | 3.835466 | -3.42777 | 0.753858 |
| C | 4.199511 | -4.25806 | 1.804642 |
| C | 3.511217 | -4.23209 | 3.02142 |
| C | 2.449793 | -3.33677 | 3.143761 |
| C | 2.081849 | -2.50024 | 2.093573 |
| C | 1.993195 | -3.53396 | -1.80954 |
| C | 1.949187 | -4.12255 | -3.06705 |
| C | 2.400752 | -3.44397 | -4.20167 |
| C | 2.890539 | -2.14901 | -4.02882 |
| C | 2.934052 | -1.55379 | -2.77262 |
| S | -7.80883 | -0.61612 | 0.101236 |
| C | -8.9888 | -1.91889 | 0.003529 |
| C | -8.29567 | -3.10909 | -0.14141 |
| C | -6.90447 | -3.00791 | -0.1601 |
| S | 7.806377 | 0.622176 | -0.12157 |
| C | 8.986274 | 1.920518 | 0.024145 |
| C | 8.29399 | 3.101247 | 0.235353 |
| C | 6.903268 | 2.997101 | 0.2676 |
| C | -8.67571 | -4.53468 | -0.31082 |
| N | -7.46019 | -5.21436 | -0.39635 |
| C | -6.347 | -4.36888 | -0.31957 |
| C | 8.675011 | 4.517402 | 0.469401 |
| N | 7.460143 | 5.190328 | 0.603478 |
| C | 6.346752 | 4.34809 | 0.499305 |
| C | -10.4085 | -1.88484 | 0.105115 |
| C | 10.40454 | 1.894013 | -0.09769 |
| C | -11.3003 | -0.84751 | 0.167116 |
| C | -10.974 | 0.583619 | -0.01763 |
| O | -9.87022 | 1.09918 | -0.05277 |
| O | -5.19859 | -4.75 | -0.38141 |
| O | -9.77379 | -5.03865 | -0.3797 |
| O | 9.773453 | 5.019028 | 0.548645 |
| O | 5.198877 | 4.724517 | 0.592111 |
| C | 11.29676 | 0.862818 | -0.22298 |
| C | 10.97482 | -0.57646 | -0.10675 |
| O | 9.87232 | -1.09537 | -0.08474 |
| C | -7.35454 | -6.64413 | -0.56741 |
| C | 7.355501 | 6.610229 | 0.843539 |
| C | -3.59026 | 3.801032 | 5.623546 |
| C | -2.67933 | 5.458098 | -4.05458 |
| C | 3.891679 | -5.15505 | 4.14498 |
| C | 2.384418 | -4.09829 | -5.5547 |
| H | 5.508125 | -0.95012 | -0.29216 |
| H | 0.307155 | 2.468482 | 0.353228 |
| H | -0.30959 | -2.46929 | -0.32146 |
| H | -5.51085 | 0.951191 | 0.306309 |
| H | -4.36804 | 3.353066 | 1.044958 |
| H | -4.89388 | 4.245173 | 3.267111 |
| H | -1.61575 | 2.046934 | 4.927266 |
| H | -1.09785 | 1.137632 | 2.711942 |
| H | -1.6577 | 4.180597 | 0.316219 |
| H | -1.68737 | 5.79121 | -1.53295 |
| H | -3.53656 | 2.866231 | -4.05508 |
| H | -3.48562 | 1.244385 | -2.21749 |
| H | 4.381759 | -3.48287 | -0.18217 |
| H | 5.034167 | -4.9412 | 1.676779 |
| H | 1.896502 | -3.2891 | 4.076979 |
| H | 1.253523 | -1.81422 | 2.228276 |
| H | 1.649492 | -4.09481 | -0.9468 |
| H | 1.558299 | -5.13087 | -3.16746 |
| H | 3.245037 | -1.59265 | -4.89144 |
| H | 3.315918 | -0.54332 | -2.67686 |
| H | -10.8144 | -2.89379 | 0.104271 |
| H | 10.80924 | 2.902439 | -0.05209 |
| H | -8.36676 | -7.04583 | -0.58313 |
| H | -6.84706 | -6.8805 | -1.50439 |
| H | -6.79287 | -7.08433 | 0.258077 |
| H | 8.367988 | 7.010119 | 0.877595 |
| H | 6.793491 | 7.090704 | 0.040975 |
| H | 6.848905 | 6.800745 | 1.791304 |
| H | -3.14011 | 4.793213 | 5.73477 |
| H | -4.6657 | 3.914351 | 5.779797 |
| H | -3.19634 | 3.171885 | 6.424419 |
| H | -1.84196 | 6.158102 | -4.00857 |
| H | -2.65896 | 4.96886 | -5.03084 |
| H | -3.60027 | 6.048834 | -4.00474 |
| H | 4.97701 | -5.22656 | 4.252164 |
| H | 3.479613 | -4.81555 | 5.097331 |
| H | 3.516891 | -6.16841 | 3.966786 |
| H | 1.506854 | -4.73728 | -5.67867 |
| H | 2.379634 | -3.35691 | -6.35647 |
| H | 3.268103 | -4.72926 | -5.6976 |
| C | -12.262 | 1.284544 | -0.18556 |
| C | -13.3169 | 0.373613 | -0.04782 |
| C | -14.6259 | 0.825291 | -0.19218 |
| H | -15.4829 | 0.172389 | -0.10497 |
| C | -12.7569 | -0.95877 | 0.263537 |
| C | -13.4884 | -2.0501 | 0.663874 |
| C | -12.9109 | -3.26833 | 1.128913 |
| N | -12.4945 | -4.2694 | 1.546469 |
| C | -14.9133 | -2.04594 | 0.715016 |
| N | -16.0742 | -2.0715 | 0.75898 |
| C | 12.26593 | -1.28217 | 0.010226 |
| C | 13.31768 | -0.36319 | -0.09444 |
| C | 14.62903 | -0.81849 | 0.011743 |
| H | 15.48382 | -0.16008 | -0.0533 |
| C | 12.75188 | 0.981873 | -0.33205 |
| C | 13.47654 | 2.093318 | -0.68677 |
| C | 12.89138 | 3.331994 | -1.08322 |
| N | 12.46848 | 4.351788 | -1.44526 |
| C | 14.9007 | 2.094718 | -0.7561 |
| N | 16.06093 | 2.124874 | -0.81348 |
| C | -12.4648 | 2.624945 | -0.4494 |
| H | -11.6257 | 3.303315 | -0.54357 |
| C | -13.7723 | 3.078114 | -0.59394 |
| C | -14.844 | 2.175208 | -0.46891 |
| C | 12.47421 | -2.63357 | 0.204583 |
| H | 11.63745 | -3.31779 | 0.27446 |
| C | 13.7841 | -3.09036 | 0.311251 |
| C | 14.85266 | -2.17996 | 0.218704 |
| Cl | -16.4707 | 2.71439 | -0.65543 |
| Cl | -14.0465 | 4.746536 | -0.92887 |
| Cl | 14.06497 | -4.77281 | 0.559394 |
| Cl | 16.48235 | -2.72406 | 0.359317 |

**Table S4:** Cartesian coordinates of **TPD3**

| **Atom** | **X-axis** | **Y-axis** | **Z-axis** |
| --- | --- | --- | --- |
| C | 2.709776 | 0.697568 | 0.038944 |
| C | 3.373548 | -0.5024 | -0.13594 |
| C | 4.762642 | -0.35386 | -0.13967 |
| C | 5.156754 | 0.970885 | 0.026391 |
| S | 3.766265 | 2.034748 | 0.196899 |
| C | 1.281896 | 0.498458 | 0.056586 |
| C | 1.064179 | -0.89212 | -0.0963 |
| C | 2.389648 | -1.65808 | -0.22917 |
| C | 0.221114 | 1.400974 | 0.178574 |
| C | -1.0635 | 0.890964 | 0.154493 |
| C | -1.28116 | -0.49825 | -0.0106 |
| C | -0.22075 | -1.3996 | -0.14216 |
| C | -2.39027 | 1.660013 | 0.255517 |
| C | -3.37439 | 0.508991 | 0.116591 |
| C | -2.70926 | -0.69517 | -0.01895 |
| C | -4.76332 | 0.358233 | 0.128316 |
| C | -5.15581 | -0.97042 | -0.00767 |
| S | -3.76378 | -2.03766 | -0.1398 |
| C | 6.503154 | 1.453207 | 0.061998 |
| C | -6.50173 | -1.4542 | -0.04042 |
| C | 2.409135 | -2.36666 | -1.58911 |
| C | 2.661591 | -2.6143 | 0.939522 |
| C | -2.61194 | 2.320549 | 1.62275 |
| C | -2.46302 | 2.66547 | -0.90021 |
| C | -3.65593 | 3.237833 | 1.785656 |
| C | -3.93362 | 3.791101 | 3.027983 |
| C | -3.18109 | 3.453213 | 4.156588 |
| C | -2.14625 | 2.533787 | 3.990961 |
| C | -1.86481 | 1.974623 | 2.747658 |
| C | -1.91514 | 3.944113 | -0.76237 |
| C | -1.89987 | 4.832385 | -1.83061 |
| C | -2.43297 | 4.481529 | -3.07305 |
| C | -2.97538 | 3.20274 | -3.20664 |
| C | -2.9899 | 2.308505 | -2.14188 |
| C | 3.69052 | -3.55711 | 0.838755 |
| C | 4.016729 | -4.3756 | 1.911434 |
| C | 3.330052 | -4.28762 | 3.125718 |
| C | 2.309899 | -3.34212 | 3.223725 |
| C | 1.979925 | -2.5179 | 2.151822 |
| C | 1.862293 | -3.64518 | -1.73109 |
| C | 1.798146 | -4.25939 | -2.97588 |
| C | 2.278857 | -3.62468 | -4.12324 |
| C | 2.819145 | -2.34611 | -3.97714 |
| C | 2.883008 | -1.72568 | -2.73452 |
| S | -7.82689 | -0.31167 | 0.056574 |
| C | -9.05569 | -1.56938 | -0.02396 |
| C | -8.40866 | -2.78754 | -0.14284 |
| C | -7.01384 | -2.74033 | -0.15535 |
| S | 7.826628 | 0.314824 | -0.09082 |
| C | 9.056933 | 1.567989 | 0.027819 |
| C | 8.411796 | 2.781157 | 0.197202 |
| C | 7.017059 | 2.734134 | 0.21922 |
| C | -8.84216 | -4.20012 | -0.28985 |
| N | -7.65305 | -4.92736 | -0.35654 |
| C | -6.50851 | -4.12408 | -0.28773 |
| C | 8.847592 | 4.186907 | 0.394032 |
| N | 7.659621 | 4.911759 | 0.497151 |
| C | 6.513904 | 4.112292 | 0.406783 |
| C | -10.4745 | -1.47749 | 0.069107 |
| C | 10.47508 | 1.478855 | -0.07771 |
| C | -11.3241 | -0.40556 | 0.10679 |
| C | -10.9417 | 1.009226 | -0.09965 |
| O | -9.818 | 1.479799 | -0.13812 |
| O | -5.3753 | -4.55036 | -0.33588 |
| O | -9.9582 | -4.66335 | -0.35667 |
| O | 9.964515 | 4.646713 | 0.469442 |
| O | 5.38143 | 4.537273 | 0.478224 |
| C | 11.32364 | 0.408608 | -0.16057 |
| C | 10.9411 | -1.01278 | -0.00623 |
| O | 9.817301 | -1.48413 | 0.016865 |
| C | -7.60162 | -6.36274 | -0.50283 |
| C | 7.610483 | 6.340783 | 0.696762 |
| C | -3.46381 | 4.080457 | 5.492873 |
| C | -2.44824 | 5.457632 | -4.21587 |
| C | 3.664531 | -5.19907 | 4.273084 |
| C | 2.241752 | -4.30629 | -5.46236 |
| H | 5.469494 | -1.16645 | -0.25291 |
| H | 0.400565 | 2.465311 | 0.282851 |
| H | -0.40117 | -2.45974 | -0.28153 |
| H | -5.47124 | 1.170841 | 0.234924 |
| H | -4.25089 | 3.531859 | 0.927008 |
| H | -4.7503 | 4.500815 | 3.122668 |
| H | -1.54533 | 2.246165 | 4.848615 |
| H | -1.05423 | 1.260472 | 2.659873 |
| H | -1.50754 | 4.254357 | 0.194002 |
| H | -1.46706 | 5.81909 | -1.69407 |
| H | -3.39439 | 2.897271 | -4.16086 |
| H | -3.41381 | 1.320454 | -2.28367 |
| H | 4.234871 | -3.66016 | -0.0943 |
| H | 4.82016 | -5.09831 | 1.802706 |
| H | 1.759658 | -3.24486 | 4.154926 |
| H | 1.183624 | -1.79163 | 2.267577 |
| H | 1.493899 | -4.17276 | -0.85765 |
| H | 1.367604 | -5.25325 | -3.05558 |
| H | 3.19718 | -1.82312 | -4.85064 |
| H | 3.303332 | -0.72881 | -2.65948 |
| H | -10.92 | -2.46944 | 0.086462 |
| H | 10.92106 | 2.47058 | -0.05996 |
| H | -8.62826 | -6.72625 | -0.50978 |
| H | -7.10564 | -6.6346 | -1.43641 |
| H | -7.05467 | -6.80901 | 0.329231 |
| H | 8.637736 | 6.702081 | 0.717458 |
| H | 7.064682 | 6.819067 | -0.11814 |
| H | 7.11462 | 6.578151 | 1.639708 |
| H | -2.9908 | 5.064885 | 5.573805 |
| H | -4.53596 | 4.222993 | 5.648092 |
| H | -3.08217 | 3.466129 | 6.310999 |
| H | -1.58308 | 6.123863 | -4.18384 |
| H | -2.44485 | 4.94381 | -5.17961 |
| H | -3.34434 | 6.086625 | -4.18445 |
| H | 4.739994 | -5.38119 | 4.337829 |
| H | 3.334369 | -4.77948 | 5.225611 |
| H | 3.177136 | -6.17295 | 4.157422 |
| H | 1.396857 | -4.99459 | -5.53675 |
| H | 2.162356 | -3.58264 | -6.27665 |
| H | 3.153083 | -4.88949 | -5.63248 |
| C | -12.1983 | 1.75855 | -0.28283 |
| C | -13.2899 | 0.892397 | -0.13452 |
| C | -14.5862 | 1.390543 | -0.28344 |
| H | -15.4782 | 0.786933 | -0.18917 |
| C | -12.7851 | -0.4559 | 0.197064 |
| C | -13.5623 | -1.51223 | 0.60529 |
| C | -13.0388 | -2.74816 | 1.087196 |
| N | -12.6684 | -3.76152 | 1.518256 |
| C | -14.9861 | -1.44884 | 0.647047 |
| N | -16.1474 | -1.42407 | 0.682933 |
| C | 12.19782 | -1.76871 | 0.145978 |
| C | 13.28945 | -0.89812 | 0.026271 |
| C | 14.58589 | -1.40168 | 0.154109 |
| H | 15.4779 | -0.79534 | 0.079128 |
| C | 12.78418 | 0.461438 | -0.25491 |
| C | 13.56027 | 1.531281 | -0.62853 |
| C | 13.03522 | 2.783421 | -1.06464 |
| N | 12.66298 | 3.81115 | -1.45852 |
| C | 14.9839 | 1.468748 | -0.67829 |
| N | 16.14499 | 1.444657 | -0.71974 |
| C | -12.3395 | 3.108121 | -0.56603 |
| H | -11.4802 | 3.759248 | -0.67042 |
| C | -13.626 | 3.590231 | -0.71237 |
| C | -14.7281 | 2.736502 | -0.57524 |
| C | 12.33916 | -3.12761 | 0.380126 |
| H | 11.47986 | -3.78183 | 0.462884 |
| C | 13.62585 | -3.61499 | 0.506429 |
| C | 14.72798 | -2.75722 | 0.397438 |
| F | 15.9406 | -3.28244 | 0.53495 |
| F | 13.85008 | -4.90422 | 0.736558 |
| F | -13.8501 | 4.8703 | -0.98905 |
| F | -15.9406 | 3.255946 | -0.73426 |

**Table S5:** Cartesian coordinates of **TPD4**

| **Atom** | **X-axis** | **Y-axis** | **Z-axis** |
| --- | --- | --- | --- |
| C | 2.689448 | 0.764242 | 0.043612 |
| C | 3.383502 | -0.41634 | -0.15497 |
| C | 4.766746 | -0.23188 | -0.15953 |
| C | 5.127251 | 1.100909 | 0.029479 |
| S | 3.70994 | 2.125428 | 0.222579 |
| C | 1.268077 | 0.52833 | 0.061726 |
| C | 1.085816 | -0.86503 | -0.11514 |
| C | 2.42937 | -1.5949 | -0.2661 |
| C | 0.185736 | 1.402284 | 0.202885 |
| C | -1.08552 | 0.86088 | 0.173608 |
| C | -1.26786 | -0.53085 | -0.01538 |
| C | -0.18591 | -1.40354 | -0.16595 |
| C | -2.43035 | 1.594821 | 0.291577 |
| C | -3.38515 | 0.421866 | 0.135011 |
| C | -2.6897 | -0.76392 | -0.02301 |
| C | -4.76813 | 0.235349 | 0.149058 |
| C | -5.12719 | -1.10195 | -0.0079 |
| S | -3.70848 | -2.13096 | -0.1626 |
| C | 6.458806 | 1.615695 | 0.070303 |
| C | -6.45849 | -1.61773 | -0.0436 |
| C | 2.462709 | -2.27777 | -1.63895 |
| C | 2.730299 | -2.56423 | 0.884801 |
| C | -2.66487 | 2.223631 | 1.671776 |
| C | -2.53159 | 2.618677 | -0.84571 |
| C | -3.72521 | 3.118439 | 1.852351 |
| C | -4.01429 | 3.639461 | 3.106029 |
| C | -3.25749 | 3.290462 | 4.228415 |
| C | -2.20625 | 2.393172 | 4.04474 |
| C | -1.91324 | 1.86629 | 2.790137 |
| C | -2.01199 | 3.906665 | -0.68628 |
| C | -2.02233 | 4.814769 | -1.73766 |
| C | -2.55359 | 4.475132 | -2.98414 |
| C | -3.06691 | 3.186848 | -3.13936 |
| C | -3.05579 | 2.272693 | -2.09153 |
| C | 3.782714 | -3.47849 | 0.763767 |
| C | 4.133826 | -4.30672 | 1.820784 |
| C | 3.44974 | -4.25744 | 3.039068 |
| C | 2.406051 | -3.34053 | 3.156898 |
| C | 2.051026 | -2.50629 | 2.100686 |
| C | 1.949188 | -3.56796 | -1.80193 |
| C | 1.89854 | -4.16174 | -3.05679 |
| C | 2.359767 | -3.49447 | -4.19422 |
| C | 2.866062 | -2.20503 | -4.02701 |
| C | 2.916346 | -1.60461 | -2.77361 |
| S | -7.81227 | -0.5125 | 0.076076 |
| C | -9.00834 | -1.80235 | -0.01903 |
| C | -8.32733 | -3.00238 | -0.15984 |
| C | -6.93777 | -2.91774 | -0.17685 |
| S | 7.811487 | 0.516405 | -0.10401 |
| C | 9.008353 | 1.801795 | 0.032191 |
| C | 8.328577 | 2.995449 | 0.223781 |
| C | 6.939166 | 2.909993 | 0.248063 |
| C | -8.72488 | -4.42486 | -0.32588 |
| N | -7.51857 | -5.11925 | -0.4081 |
| C | -6.39581 | -4.28661 | -0.33176 |
| C | 8.727631 | 4.410489 | 0.441634 |
| N | 7.5221 | 5.10111 | 0.559173 |
| C | 6.398629 | 4.271973 | 0.45881 |
| C | -10.4237 | -1.75397 | 0.080235 |
| C | 10.42301 | 1.757492 | -0.07826 |
| C | -11.3073 | -0.70492 | 0.144823 |
| C | -10.9641 | 0.718036 | -0.03633 |
| O | -9.85834 | 1.227595 | -0.08061 |
| O | -5.25176 | -4.67956 | -0.39036 |
| O | -9.83007 | -4.91243 | -0.39419 |
| O | 9.833398 | 4.895188 | 0.520374 |
| O | 5.255108 | 4.662571 | 0.539334 |
| C | 11.30614 | 0.711884 | -0.1882 |
| C | 10.96412 | -0.71707 | -0.05975 |
| O | 9.858791 | -1.22853 | -0.03125 |
| C | -7.42942 | -6.55116 | -0.57545 |
| C | 7.434434 | 6.525713 | 0.780878 |
| C | -3.55312 | 3.883483 | 5.577344 |
| C | -2.59762 | 5.472216 | -4.10778 |
| C | 3.813815 | -5.17908 | 4.168997 |
| C | 2.336501 | -4.15477 | -5.54415 |
| H | 5.493731 | -1.02407 | -0.28905 |
| H | 0.339203 | 2.468796 | 0.324665 |
| H | -0.3405 | -2.46526 | -0.32299 |
| H | -5.49578 | 1.028002 | 0.271961 |
| H | -4.32365 | 3.421141 | 0.999164 |
| H | -4.84311 | 4.332869 | 3.214585 |
| H | -1.60162 | 2.097607 | 4.897026 |
| H | -1.09017 | 1.168295 | 2.68882 |
| H | -1.60753 | 4.208678 | 0.274087 |
| H | -1.61214 | 5.808595 | -1.58438 |
| H | -3.48357 | 2.88988 | -4.09723 |
| H | -3.45761 | 1.278062 | -2.25081 |
| H | 4.325148 | -3.55235 | -0.1732 |
| H | 4.954572 | -5.00706 | 1.696412 |
| H | 1.856867 | -3.27406 | 4.091363 |
| H | 1.236656 | -1.80308 | 2.232303 |
| H | 1.597829 | -4.12053 | -0.93694 |
| H | 1.495009 | -5.16544 | -3.15277 |
| H | 3.228171 | -1.65733 | -4.89196 |
| H | 3.310706 | -0.59844 | -2.68289 |
| H | -10.8413 | -2.75824 | 0.075072 |
| H | 10.84066 | 2.76092 | -0.03718 |
| H | -8.44601 | -6.94156 | -0.58945 |
| H | -6.92547 | -6.79535 | -1.51222 |
| H | -6.87227 | -6.99495 | 0.251031 |
| H | 8.451544 | 6.913687 | 0.813044 |
| H | 6.880828 | 7.002188 | -0.02971 |
| H | 6.927633 | 6.734182 | 1.724645 |
| H | -3.09633 | 4.873419 | 5.680585 |
| H | -4.6277 | 4.005452 | 5.732944 |
| H | -3.16322 | 3.257851 | 6.382848 |
| H | -1.74396 | 6.152867 | -4.07174 |
| H | -2.59577 | 4.977058 | -5.08117 |
| H | -3.50406 | 6.084586 | -4.0546 |
| H | 4.896672 | -5.30011 | 4.252167 |
| H | 3.440545 | -4.80589 | 5.124754 |
| H | 3.387584 | -6.17617 | 4.016469 |
| H | 1.457862 | -4.79342 | -5.66124 |
| H | 2.329109 | -3.41707 | -6.34928 |
| H | 3.218979 | -4.78717 | -5.68788 |
| C | -12.2472 | 1.440479 | -0.19103 |
| C | -13.3126 | 0.541419 | -0.05163 |
| C | -14.6185 | 1.007003 | -0.18674 |
| H | -15.4805 | 0.360764 | -0.09813 |
| C | -12.7621 | -0.8014 | 0.246892 |
| C | -13.5081 | -1.88397 | 0.643825 |
| C | -12.9438 | -3.11231 | 1.098129 |
| N | -12.5363 | -4.12059 | 1.506793 |
| C | -14.9326 | -1.86216 | 0.700769 |
| N | -16.0934 | -1.87221 | 0.749297 |
| C | 12.24824 | -1.44398 | 0.063411 |
| C | 13.31267 | -0.53978 | -0.04677 |
| C | 14.61924 | -1.00903 | 0.067103 |
| H | 15.48058 | -0.35939 | -0.00107 |
| C | 12.7604 | 0.812745 | -0.29373 |
| C | 13.50397 | 1.909442 | -0.6548 |
| C | 12.93658 | 3.15299 | -1.0615 |
| N | 12.52619 | 4.174974 | -1.43133 |
| C | 14.92812 | 1.890461 | -0.71967 |
| N | 16.08873 | 1.902698 | -0.77368 |
| C | -12.4278 | 2.785368 | -0.44599 |
| H | -11.5762 | 3.448286 | -0.54054 |
| C | -13.7338 | 3.261027 | -0.58201 |
| C | -14.8223 | 2.365369 | -0.45562 |
| C | 12.43039 | -2.79705 | 0.269046 |
| H | 11.57954 | -3.46358 | 0.342184 |
| C | 13.73709 | -3.27637 | 0.384086 |
| C | 14.82472 | -2.37603 | 0.286519 |
| C | 16.1643 | -2.85976 | 0.414736 |
| N | 17.25606 | -3.23738 | 0.518216 |
| C | 13.96095 | -4.67032 | 0.603315 |
| N | 14.10992 | -5.80693 | 0.781174 |
| C | -13.9562 | 4.646429 | -0.8512 |
| N | -14.1043 | 5.776098 | -1.06939 |
| C | -16.1612 | 2.844981 | -0.60502 |
| N | -17.2524 | 3.219233 | -0.72508 |

**Table S6:** Cartesian coordinates of **TPD5**

| **Atom** | **X-axis** | **Y-axis** | **Z-axis** |
| --- | --- | --- | --- |
| C | 2.625106 | 0.940264 | 0.143014 |
| C | 3.40037 | -0.18174 | -0.09171 |
| C | 4.766302 | 0.104231 | -0.11106 |
| C | 5.030865 | 1.45552 | 0.10411 |
| S | 3.545602 | 2.368077 | 0.342298 |
| C | 1.22512 | 0.601452 | 0.170111 |
| C | 1.141653 | -0.797 | -0.03826 |
| C | 2.532616 | -1.42451 | -0.21606 |
| C | 0.084599 | 1.392403 | 0.339505 |
| C | -1.14511 | 0.762857 | 0.304247 |
| C | -1.22935 | -0.6332 | 0.081007 |
| C | -0.08906 | -1.42311 | -0.09566 |
| C | -2.53747 | 1.396863 | 0.444811 |
| C | -3.40816 | 0.164792 | 0.254371 |
| C | -2.63136 | -0.96521 | 0.067677 |
| C | -4.77478 | -0.11806 | 0.260188 |
| C | -5.0393 | -1.47278 | 0.068082 |
| S | -3.55209 | -2.39587 | -0.10983 |
| C | 6.32072 | 2.067543 | 0.135028 |
| C | -6.331 | -2.07932 | 0.013592 |
| C | 2.600236 | -2.07965 | -1.60101 |
| C | 2.917849 | -2.38977 | 0.913025 |
| C | -2.81096 | 1.963201 | 1.844783 |
| C | -2.71262 | 2.444839 | -0.66074 |
| C | -3.93076 | 2.774757 | 2.056351 |
| C | -4.25263 | 3.230156 | 3.327591 |
| C | -3.47094 | 2.895613 | 4.437135 |
| C | -2.35963 | 2.081352 | 4.22229 |
| C | -2.03324 | 1.620841 | 2.950081 |
| C | -2.30097 | 3.765007 | -0.4575 |
| C | -2.37887 | 4.701777 | -1.48086 |
| C | -2.87177 | 4.358954 | -2.74183 |
| C | -3.27519 | 3.037843 | -2.94159 |
| C | -3.19693 | 2.095786 | -1.92202 |
| C | 4.054625 | -3.19448 | 0.774389 |
| C | 4.478467 | -4.01595 | 1.809186 |
| C | 3.785768 | -4.06873 | 3.022967 |
| C | 2.657565 | -3.2616 | 3.158317 |
| C | 2.229023 | -2.43403 | 2.123745 |
| C | 2.207153 | -3.40903 | -1.77744 |
| C | 2.185737 | -3.98376 | -3.04261 |
| C | 2.556697 | -3.25722 | -4.17587 |
| C | 2.941995 | -1.92771 | -3.99499 |
| C | 2.963064 | -1.34699 | -2.73213 |
| S | -7.75942 | -1.07694 | 0.167737 |
| C | -8.86104 | -2.44353 | 0.021492 |
| C | -8.09702 | -3.5875 | -0.15617 |
| C | -6.71688 | -3.40485 | -0.16425 |
| S | 7.746994 | 1.083901 | -0.12378 |
| C | 8.846584 | 2.449417 | 0.045832 |
| C | 8.084327 | 3.578092 | 0.308628 |
| C | 6.706471 | 3.385936 | 0.360458 |
| C | -8.39291 | -5.0279 | -0.37104 |
| N | -7.14052 | -5.63262 | -0.4717 |
| C | -6.07932 | -4.72605 | -0.36276 |
| C | 8.381381 | 5.00791 | 0.584012 |
| N | 7.13089 | 5.598586 | 0.761894 |
| C | 6.070583 | 4.691684 | 0.647307 |
| C | -10.277 | -2.4983 | 0.109103 |
| C | 10.25679 | 2.519477 | -0.10188 |
| C | -11.2324 | -1.51679 | 0.201807 |
| C | -10.9893 | -0.06504 | 0.082021 |
| O | -9.91962 | 0.517234 | 0.079588 |
| O | -4.91027 | -5.03541 | -0.4293 |
| O | -9.46078 | -5.59004 | -0.4591 |
| O | 9.449142 | 5.571915 | 0.660432 |
| O | 4.903345 | 4.990205 | 0.770903 |
| C | 11.21263 | 1.55174 | -0.28799 |
| C | 10.98492 | 0.093866 | -0.22523 |
| O | 9.920296 | -0.49657 | -0.1899 |
| C | -6.95081 | -7.04863 | -0.6841 |
| C | 6.942576 | 7.001389 | 1.049789 |
| C | -3.80389 | 3.418931 | 5.80608 |
| C | -2.99125 | 5.383587 | -3.8348 |
| C | 4.233923 | -4.98223 | 4.129038 |
| C | 2.569459 | -3.89316 | -5.53756 |
| H | 5.54785 | -0.62907 | -0.26744 |
| H | 0.163223 | 2.464254 | 0.48397 |
| H | -0.16906 | -2.4889 | -0.27914 |
| H | -5.55635 | 0.618561 | 0.399714 |
| H | -4.55078 | 3.064466 | 1.214179 |
| H | -5.12784 | 3.859345 | 3.460312 |
| H | -1.73394 | 1.798982 | 5.063796 |
| H | -1.16354 | 0.986237 | 2.824259 |
| H | -1.9283 | 4.068915 | 0.515019 |
| H | -2.05284 | 5.720591 | -1.2934 |
| H | -3.65868 | 2.737527 | -3.91219 |
| H | -3.5142 | 1.076924 | -2.11562 |
| H | 4.606533 | -3.18719 | -0.15999 |
| H | 5.363881 | -4.62965 | 1.671616 |
| H | 2.099136 | -3.27705 | 4.08951 |
| H | 1.349223 | -1.81784 | 2.268959 |
| H | 1.927734 | -4.00688 | -0.91643 |
| H | 1.877366 | -5.01956 | -3.14946 |
| H | 3.231605 | -1.33341 | -4.85656 |
| H | 3.262556 | -0.30958 | -2.62983 |
| H | -10.6224 | -3.52851 | 0.062258 |
| H | 10.59813 | 3.548933 | -0.02134 |
| H | -7.93791 | -7.50686 | -0.72569 |
| H | -6.41935 | -7.22589 | -1.62064 |
| H | -6.37506 | -7.48128 | 0.135629 |
| H | 7.929141 | 7.46091 | 1.089611 |
| H | 6.345368 | 7.472824 | 0.267579 |
| H | 6.434096 | 7.128652 | 2.006914 |
| H | -3.39729 | 4.425292 | 5.95209 |
| H | -4.88405 | 3.481221 | 5.957794 |
| H | -3.38793 | 2.781893 | 6.589384 |
| H | -2.21627 | 6.148818 | -3.75142 |
| H | -2.91028 | 4.925005 | -4.82272 |
| H | -3.95915 | 5.894088 | -3.79046 |
| H | 5.319837 | -4.95815 | 4.250434 |
| H | 3.780874 | -4.70626 | 5.08324 |
| H | 3.955188 | -6.02025 | 3.91988 |
| H | 1.823054 | -4.68688 | -5.61451 |
| H | 2.368172 | -3.16087 | -6.32256 |
| H | 3.545263 | -4.34147 | -5.75263 |
| C | -12.3176 | 0.569242 | -0.06821 |
| C | -13.317 | -0.40903 | 0.017438 |
| C | -14.657 | -0.04084 | -0.12586 |
| H | -15.463 | -0.75926 | -0.07199 |
| C | -12.6765 | -1.71909 | 0.279094 |
| C | -13.3495 | -2.86418 | 0.632646 |
| C | -12.7021 | -4.06101 | 1.059401 |
| N | -12.2277 | -5.04876 | 1.445483 |
| C | -14.7719 | -2.95253 | 0.670943 |
| N | -15.9284 | -3.05977 | 0.706596 |
| C | 12.32313 | -0.53588 | -0.18076 |
| C | 13.31049 | 0.453913 | -0.27191 |
| C | 14.65853 | 0.090532 | -0.22023 |
| H | 15.45586 | 0.818886 | -0.26904 |
| C | 12.64948 | 1.770377 | -0.4287 |
| C | 13.29709 | 2.93858 | -0.75333 |
| C | 12.62253 | 4.15143 | -1.08137 |
| N | 12.12457 | 5.155176 | -1.38856 |
| C | 14.71527 | 3.041938 | -0.85548 |
| N | 15.86795 | 3.162585 | -0.93889 |
| C | -12.61 | 1.902755 | -0.27459 |
| H | -11.8266 | 2.649303 | -0.32698 |
| C | -13.9467 | 2.315738 | -0.43174 |
| C | -14.9585 | 1.302665 | -0.3556 |
| C | 12.63465 | -1.87586 | -0.06181 |
| H | 11.85973 | -2.63078 | -0.00364 |
| C | 13.98046 | -2.28632 | -0.01394 |
| C | 14.9805 | -1.26122 | -0.08862 |
| C | 16.37091 | -1.58957 | -0.00439 |
| N | 17.5077 | -1.80577 | 0.079388 |
| C | 14.19973 | -3.6942 | 0.14578 |
| C | -14.1511 | 3.722507 | -0.61839 |
| C | -16.3418 | 1.641291 | -0.49725 |
| N | -17.4748 | 1.871575 | -0.59394 |
| C | -15.3853 | 4.270538 | -1.05135 |
| O | -15.4981 | 4.223756 | -2.27553 |
| O | -16.1947 | 4.897275 | -0.21024 |
| C | 15.47761 | -4.29427 | 0.009901 |
| O | 15.74624 | -4.52551 | -1.168 |
| O | 16.17329 | -4.68637 | 1.067142 |
| C | -17.3556 | 5.484903 | -0.81273 |
| H | -18.0164 | 4.703071 | -1.18971 |
| H | -17.0707 | 6.150901 | -1.62775 |
| H | -17.8442 | 6.041275 | -0.01613 |
| C | 17.4032 | -5.35911 | 0.764904 |
| H | 18.10485 | -4.66261 | 0.303753 |
| H | 17.22676 | -6.20126 | 0.095102 |
| H | 17.7865 | -5.70467 | 1.722231 |

**Table S7:** Cartesian coordinates of **TPD6**

| **Atom** | **X-axis** | **Y-axis** | **Z-axis** |
| --- | --- | --- | --- |
| C | -5.27072 | 0.783953 | 0.397512 |
| C | -4.552 | 1.967676 | 0.287808 |
| C | -3.1688 | 1.743711 | 0.316026 |
| C | -2.83205 | 0.41112 | 0.446679 |
| H | -5.04854 | 2.926931 | 0.200023 |
| C | -1.91778 | 2.608943 | 0.278441 |
| C | -0.83444 | 1.523604 | 0.36804 |
| C | 0.538449 | 1.683542 | 0.339128 |
| C | 1.329927 | 0.534105 | 0.423148 |
| C | 0.759894 | -0.75805 | 0.52288 |
| C | -0.6125 | -0.91871 | 0.550926 |
| C | -1.40323 | 0.230964 | 0.468349 |
| H | 0.986454 | 2.667213 | 0.25125 |
| H | -1.06021 | -1.90266 | 0.637028 |
| S | -4.20658 | -0.60757 | 0.544121 |
| C | 2.761164 | 0.356313 | 0.439443 |
| C | 1.841682 | -1.84546 | 0.611928 |
| C | 3.090612 | -0.97991 | 0.563602 |
| C | 4.471477 | -1.19388 | 0.585517 |
| C | 5.194678 | -0.00941 | 0.486604 |
| S | 4.128987 | 1.382694 | 0.360653 |
| H | 4.94309 | -2.16489 | 0.674331 |
| C | 0.602048 | -4.33583 | 3.243743 |
| C | 0.825409 | -3.70321 | 2.026837 |
| C | 1.673013 | -2.59515 | 1.938984 |
| C | 2.281466 | -2.14164 | 3.1101 |
| C | 2.056719 | -2.78008 | 4.324675 |
| C | 1.215947 | -3.89025 | 4.416474 |
| H | -0.06073 | -5.19543 | 3.281076 |
| H | 0.345827 | -4.08559 | 1.132016 |
| H | 2.936554 | -1.27789 | 3.077606 |
| H | 2.546191 | -2.40552 | 5.218827 |
| C | 2.070326 | -4.47582 | -2.86492 |
| C | 1.332248 | -3.29373 | -2.91049 |
| C | 1.228211 | -2.46018 | -1.80071 |
| C | 1.861004 | -2.78477 | -0.60145 |
| C | 2.611497 | -3.96487 | -0.5542 |
| C | 2.71248 | -4.79222 | -1.66426 |
| H | 0.82695 | -3.01495 | -3.83044 |
| H | 0.649597 | -1.54679 | -1.87707 |
| H | 3.111537 | -4.2465 | 0.366844 |
| H | 3.300218 | -5.70306 | -1.59612 |
| C | 1.001498 | -4.5994 | 5.724127 |
| H | 0.000095 | -5.0316 | 5.784782 |
| H | 1.717583 | -5.41858 | 5.849256 |
| H | 1.130323 | -3.92232 | 6.571299 |
| C | 2.153891 | -5.39223 | -4.05328 |
| H | 3.147971 | -5.83636 | -4.14621 |
| H | 1.437922 | -6.2163 | -3.9659 |
| H | 1.931538 | -4.86274 | -4.98198 |
| C | -2.33183 | 2.914774 | -2.22248 |
| C | -2.10096 | 3.563715 | -3.43018 |
| C | -1.27265 | 4.684461 | -3.5048 |
| C | -0.67826 | 5.13041 | -2.3223 |
| C | -0.90845 | 4.487782 | -1.11193 |
| C | -1.74246 | 3.368555 | -1.04195 |
| H | -2.9788 | 2.044557 | -2.2019 |
| H | -2.57636 | 3.190001 | -4.3323 |
| H | -0.02582 | 5.998367 | -2.34632 |
| H | -0.44483 | 4.86995 | -0.2087 |
| C | -1.9452 | 3.54413 | 1.495147 |
| C | -2.75568 | 4.685116 | 1.465576 |
| C | -2.86117 | 5.513613 | 2.573201 |
| C | -2.1636 | 5.238669 | 3.753752 |
| C | -1.36497 | 4.096988 | 3.781107 |
| C | -1.25645 | 3.260559 | 2.672957 |
| H | -3.30531 | 4.93161 | 0.563155 |
| H | -3.49944 | 6.390688 | 2.520131 |
| H | -0.81473 | 3.851474 | 4.684593 |
| H | -0.62923 | 2.378977 | 2.735955 |
| C | -1.05164 | 5.405071 | -4.80519 |
| H | -1.7654 | 6.227 | -4.92568 |
| H | -0.04907 | 5.835852 | -4.85783 |
| H | -1.17823 | 4.735963 | -5.65908 |
| C | -2.26012 | 6.156687 | 4.93991 |
| H | -1.65917 | 7.059692 | 4.789722 |
| H | -3.29025 | 6.479616 | 5.111034 |
| H | -1.90244 | 5.671629 | 5.85031 |
| C | 6.621363 | 0.106984 | 0.470112 |
| C | 7.467213 | 1.205397 | 0.491991 |
| C | 8.836924 | 0.895225 | 0.461291 |
| C | 9.120113 | -0.45081 | 0.408275 |
| S | 7.605264 | -1.33733 | 0.414559 |
| N | 8.633304 | 3.15343 | 0.729049 |
| C | 9.604047 | 2.151339 | 0.678735 |
| O | 10.78856 | 2.335334 | 0.83056 |
| C | 7.325469 | 2.669946 | 0.628573 |
| O | 6.328997 | 3.359898 | 0.660538 |
| C | 8.95489 | 4.547416 | 0.919789 |
| H | 9.455872 | 4.696281 | 1.878159 |
| H | 9.612868 | 4.895055 | 0.12176 |
| H | 8.019225 | 5.104215 | 0.900245 |
| C | -6.69283 | 0.630932 | 0.405662 |
| C | -7.69912 | 1.586262 | 0.332109 |
| C | -9.0014 | 1.06011 | 0.342733 |
| C | -9.07072 | -0.31301 | 0.425258 |
| S | -7.43546 | -0.94401 | 0.514532 |
| N | -9.16554 | 3.336337 | 0.369776 |
| C | -9.96444 | 2.18997 | 0.430596 |
| O | -11.1644 | 2.188885 | 0.568929 |
| C | -7.7994 | 3.062713 | 0.31144 |
| O | -6.94071 | 3.920134 | 0.264842 |
| C | -9.69644 | 4.677799 | 0.413804 |
| H | -9.48422 | 5.204681 | -0.51842 |
| H | -10.7731 | 4.596119 | 0.554961 |
| H | -9.25214 | 5.233338 | 1.241138 |
| C | -10.166 | -1.22324 | 0.548516 |
| H | -9.96173 | -2.09794 | 1.158153 |
| C | -13.5566 | -1.68162 | -0.82889 |
| C | -13.0653 | -0.66416 | -1.65558 |
| C | -13.7808 | -0.16969 | -2.73374 |
| C | -15.0335 | -0.71027 | -2.98883 |
| C | -15.5353 | -1.72853 | -2.17083 |
| C | -14.815 | -2.22702 | -1.089 |
| C | -11.3974 | -1.11564 | -0.02897 |
| H | -13.358 | 0.616404 | -3.34827 |
| H | -15.627 | -0.35597 | -3.82353 |
| H | -15.239 | -3.02197 | -0.49231 |
| C | -11.7173 | -0.24973 | -1.20627 |
| O | -11.007 | 0.560208 | -1.76222 |
| C | -12.5536 | -1.9795 | 0.214041 |
| C | -12.723 | -2.86458 | 1.251938 |
| C | -13.8986 | -3.65302 | 1.422664 |
| N | -14.8388 | -4.31455 | 1.591578 |
| C | -11.7511 | -3.07877 | 2.273218 |
| N | -10.9811 | -3.28311 | 3.119601 |
| C | 10.34812 | -1.18168 | 0.456178 |
| H | 10.28852 | -2.13483 | 0.97202 |
| C | 13.12983 | 0.098558 | -1.59743 |
| C | 13.78234 | -0.90388 | -0.86919 |
| C | 15.12306 | -1.18164 | -1.13896 |
| C | 15.76218 | -0.43883 | -2.12888 |
| C | 15.09887 | 0.560198 | -2.84839 |
| C | 13.76306 | 0.83331 | -2.58558 |
| C | 11.54607 | -0.81502 | -0.08396 |
| H | 15.67289 | -1.95274 | -0.61813 |
| H | 15.63388 | 1.10928 | -3.61399 |
| H | 13.21562 | 1.59649 | -3.12622 |
| C | 11.72271 | 0.214972 | -1.1553 |
| O | 10.88644 | 0.940263 | -1.64916 |
| C | 12.83141 | -1.49199 | 0.097061 |
| C | 13.13879 | -2.45413 | 1.029496 |
| C | 12.20575 | -2.95663 | 1.983615 |
| N | 11.4718 | -3.39295 | 2.772291 |
| C | 14.43118 | -3.04446 | 1.147152 |
| N | 15.46955 | -3.55123 | 1.270498 |
| C | 17.22504 | -0.67645 | -2.39923 |
| F | 17.51703 | -0.49702 | -3.69751 |
| F | 17.98784 | 0.179583 | -1.70082 |
| F | 17.59986 | -1.91657 | -2.06281 |
| C | -16.9136 | -2.2627 | -2.46119 |
| F | -17.859 | -1.39966 | -2.05579 |
| F | -17.1407 | -3.42993 | -1.84855 |
| F | -17.0912 | -2.44901 | -3.77958 |

**Table S8**: Wavelength, excitation energy and oscillator strength of **TPDR** in gas

| **NO** | **DFT λ(nm)** | **E(eV)** | ***f*** | **MO contributions** |
| --- | --- | --- | --- | --- |
| 1 | 639.291 | 1.939 | 2.145 | H→L (97%), H-1→L+1 (2%) |
| 2 | 579.338 | 2.140 | 0.000 | H→L+1 (99%), |
| 3 | 476.240 | 2.603 | 0.894 | H→L+2 (85%), H-1→L+1 (8%) |
| 4 | 465.808 | 2.662 | 0.000 | H-1→L (98%), |
| 5 | 439.084 | 2.824 | 0.279 | H-1→L+1 (88%), H→L+2 (9%) |
| 6 | 420.385 | 2.949 | 0.022 | H-3→L (11%), H-2→L (82%), H-4→L+1 (5%) |

MO=molecular orbital, H=HOMO, L=LUMO, *f=* oscillator strength

**Table S9**: Wavelength, excitation energy and oscillator strength of **TPD1** in gas

| **NO** | **DFT λ (nm)** | **E(eV)** | ***f*** | **MO contributions** |
| --- | --- | --- | --- | --- |
| 1 | 703.736 | 1.762 | 2.038 | H→L (98%), |
| 2 | 635.979 | 1.950 | 0.000 | H→L+1 (99%), |
| 3 | 492.196 | 2.519 | 0.001 | H-1→L (98%), |
| 4 | 482.504 | 2.570 | 1.031 | H-1→L+1 (46%), H→L+2 (47%), H→L (2%) |
| 5 | 457.135 | 2.712 | 0.126 | H-1→L+1 (48%), H→L+2 (44%), H-2→L (2%) |
| 6 | 452.778 | 2.738 | 0.009 | H-2→L (90%), H-3→L+1 (6%), H-1→L+1 (2%) |

MO=molecular orbital, H=HOMO, L=LUMO, *f=* oscillator strength

**Table S10**: Wavelength, excitation energy and oscillator strength of **TPD2** in gas

| **NO** | **DFT λ (nm)** | **E(eV)** | ***f*** | **MO contributions** |
| --- | --- | --- | --- | --- |
| 1 | 714.647 | 1.735 | 2.081 | H→L (97%), |
| 2 | 644.475 | 1.924 | 0.000 | H→L+1 (99%), |
| 3 | 507.300 | 2.444 | 0.259 | H→L+2 (92%), H-1→L+3 (3%) |
| 4 | 501.656 | 2.472 | 0.000 | H→L+3 (92%), H-1→L (3%), H-1→L+2 (3%) |
| 5 | 495.263 | 2.503 | 0.000 | H-1→L (95%), H→L+3 (3%) |
| 6 | 480.969 | 2.578 | 0.753 | H-1→L+1 (57%), H→L+4 (34%), H→L+2 (3%) |

MO=molecular orbital, H=HOMO, L=LUMO, *f=* oscillator strength

**Table S11**: Wavelength, excitation energy and oscillator strength of **TPD3** in gas

| **NO** | **DFT λ (nm)** | **E(eV)** | ***f*** | **MO contributions** |
| --- | --- | --- | --- | --- |
| 1 | 701.347 | 1.768 | 2.026 | H→L (97%), |
| 2 | 634.125 | 1.955 | 0.000 | H→L+1 (99%), |
| 3 | 497.349 | 2.493 | 0.339 | H→L+2 (90%), H-1→L+1 (3%), H-1→L+3 (3%) |
| 4 | 491.299 | 2.524 | 0.000 | H-1→L (28%), H→L+3 (68%), H-1→L+2 (2%) |
| 5 | 488.011 | 2.541 | 0.000 | H-1→L (70%), H→L+3 (27%), |
| 6 | 475.308 | 2.609 | 0.693 | H-1→L+1 (49%), H→L+4 (40%), H→L+2 (5%) |

MO=molecular orbital, H=HOMO, L=LUMO, *f=* oscillator strength

**Table S12**: Wavelength, excitation energy and oscillator strength of **TPD4** in gas

| **NO** | **DFT λ (nm)** | **E(eV)** | ***f*** | **MO contributions** |
| --- | --- | --- | --- | --- |
| 1 | 761.153 | 1.629 | 2.023 | H→L (98%), |
| 2 | 686.817 | 1.805 | 0.000 | H→L+1 (99%), |
| 3 | 595.935 | 2.081 | 0.148 | H→L+2 (96%), H-1→L+3 (3%) |
| 4 | 588.691 | 2.106 | 0.000 | H→L+3 (96%), H-1→L+2 (2%) |
| 5 | 518.741 | 2.390 | 0.000 | H-1→L (98%), |
| 6 | 502.876 | 2.466 | 0.485 | H-2→L (33%), H-1→L+1 (52%), H→L+4 (10%), |

MO=molecular orbital, H=HOMO, L=LUMO, *f=* oscillator strength

**Table S13**: Wavelength, excitation energy and oscillator strength of **TPD5** in gas

| **NO** | **DFT λ (nm)** | **E(eV)** | ***f*** | **MO contributions** |
| --- | --- | --- | --- | --- |
| 1 | 1392.142 | 0.891 | 0.000 | H-2→L (39%), H-2→L+1 (43%), H-2→L+2 (7%), H-2→L+3 (4%), H-2→L+5 (4%), H-2→L+6 (3%) |
| 2 | 1389.490 | 0.892 | 0.001 | H-1→L (41%), H-1→L+1 (41%), H-1→L+2 (8%), H-1→L+3 (4%), H-1→L+5 (3%), H-1→L+6 (5%) |
| 3 | 877.454 | 1.413 | 0.972 | H→L (98%), |
| 4 | 838.865 | 1.478 | 0.000 | H→L+1 (98%), |
| 5 | 673.534 | 1.841 | 1.318 | H→L+2 (97%), H-3→L+3 (2%) |
| 6 | 624.952 | 1.984 | 0.000 | H→L+3 (93%), H-3→L (5%) |

MO=molecular orbital, H=HOMO, L=LUMO, *f=* oscillator strength

**Table S14**: Wavelength, excitation energy and oscillator strength of **TPD6** in gas

| **NO** | **DFT λ (nm)** | **E(eV)** | ***f*** | **MO contributions** |
| --- | --- | --- | --- | --- |
| 1 | 712.307 | 1.741 | 1.875 | H→L (97%), H-1→L+1 (2%) |
| 2 | 647.572 | 1.915 | 0.047 | H→L+1 (99%) |
| 3 | 505.996 | 2.450 | 0.537 | H→L+2 (80%), H-1→L+1 (9%), H-1→L+3 (3%) |
| 4 | 500.643 | 2.477 | 0.064 | H-1→L (79%), H→L+3 (11%), |
| 5 | 492.294 | 2.519 | 0.015 | H-1→L (13%), H→L+3 (81%), H-1→L+2 (2%) |
| 6 | 478.814 | 2.589 | 0.279 | H-1→L+1 (61%), H→L+2 (13%), H→L+4 (18%), |

MO=molecular orbital, H=HOMO, L=LUMO, *f=* oscillator strength

**Table S15**: Wavelength, excitation energy and oscillator strength of **TPDR** in chloroform

| **NO** | **DFT λ (nm)** | **E(eV)** | ***f*** | **MO contributions** |
| --- | --- | --- | --- | --- |
| 1 | 658.895 | 1.882 | 2.423 | H→L (95%), H-1→L+1 (3%) |
| 2 | 591.274 | 2.097 | 0.000 | H→L+1 (98%), |
| 3 | 484.389 | 2.560 | 0.756 | H→L+2 (92%), H→L (2%) |
| 4 | 473.674 | 2.618 | 0.000 | H-1→L (95%), H-4→L+1 (2%) |
| 5 | 446.436 | 2.777 | 0.546 | H-1→L+1 (90%), H-4→L (2%), H→L (2%), H→L+2 (3%) |
| 6 | 418.766 | 2.961 | 0.018 | H-2→L (89%), H-4→L (3%), H-3→L+1 (5%), H-2→L+2 (2%) |

MO=molecular orbital, H=HOMO, L=LUMO, *f=* oscillator strength

**Table S16**: Wavelength, excitation energy and oscillator strength of **TPD1** in chloroform

| **NO** | **DFT λ (nm)** | **E(eV)** | ***f*** | **MO contributions** |
| --- | --- | --- | --- | --- |
| 1 | 741.932 | 1.671 | 2.306 | H→L (96%), H-1→L+1 (3%) |
| 2 | 663.443 | 1.869 | 0.000 | H→L+1 (99%), |
| 3 | 505.790 | 2.451 | 0.000 | H-1→L (96%), |
| 4 | 491.007 | 2.525 | 1.064 | H-1→L+1 (36%), H→L+2 (55%), H→L (3%) |
| 5 | 471.333 | 2.631 | 0.124 | H-1→L+1 (57%), H→L+2 (38%), |
| 6 | 461.422 | 2.687 | 0.003 | H-2→L (90%), H-3→L+1 (6%) |

MO=molecular orbital, H=HOMO, L=LUMO, *f=* oscillator strength

**Table S17**: Wavelength, excitation energy and oscillator strength of **TPD2** in chloroform

| **NO** | **DFT λ (nm)** | **E(eV)** | ***f*** | **MO contributions** |
| --- | --- | --- | --- | --- |
| 1 | 757.109 | 1.638 | 2.315 | H→L (96%), H-1→L+1 (3%) |
| 2 | 676.437 | 1.833 | 0.000 | H→L+1 (98%), |
| 3 | 517.139 | 2.398 | 0.320 | H→L+2 (90%), H-1→L+3 (3%), H→L+4 (3%) |
| 4 | 511.676 | 2.423 | 0.000 | H-1→L (96%), |
| 5 | 509.029 | 2.436 | 0.000 | H→L+3 (94%), H-1→L+2 (3%) |
| 6 | 490.211 | 2.529 | 0.672 | H-1→L+1 (61%), H→L+4 (26%), H→L (3%), H→L+2 (4%) |

MO=molecular orbital, H=HOMO, L=LUMO, *f=* oscillator strength

**Table S18**: Wavelength, excitation energy and oscillator strength of **TPD3** in chloroform

| **NO** | **DFT λ (nm)** | **E(eV)** | ***f*** | **MO contributions** |
| --- | --- | --- | --- | --- |
| 1 | 739.630 | 1.676 | 2.275 | H→L (96%), H-1→L+1 (3%) |
| 2 | 662.415 | 1.872 | 0.000 | H→L+1 (98%), |
| 3 | 505.068 | 2.455 | 0.458 | H→L+2 (85%), H-1→L+1 (3%), H-1→L+3 (3%), H→L+4 (6%) |
| 4 | 503.387 | 2.463 | 0.000 | H-1→L (96%), |
| 5 | 494.631 | 2.507 | 0.000 | H→L+3 (94%), H-1→L+2 (4%) |
| 6 | 482.767 | 2.568 | 0.577 | H-1→L+1 (51%), H→L+4 (33%), H→L (2%), H→L+2 (8%) |

MO=molecular orbital, H=HOMO, L=LUMO, *f=* oscillator strength

**Table S19**: Wavelength, excitation energy and oscillator strength of **TPD4** in chloroform

| **NO** | **DFT λ (nm)** | **E(eV)** | ***f*** | **MO contributions** |
| --- | --- | --- | --- | --- |
| 1 | 810.778 | 1.529 | 2.254 | H→L (96%), H-1→L+1 (2%) |
| 2 | 724.122 | 1.712 | 0.000 | H→L+1 (99%), |
| 3 | 614.940 | 2.016 | 0.183 | H→L+2 (95%), H-1→L+3 (3%) |
| 4 | 605.273 | 2.048 | 0.000 | H→L+3 (96%), H-1→L+2 (3%) |
| 5 | 537.216 | 2.308 | 0.000 | H-1→L (97%), |
| 6 | 514.137 | 2.412 | 0.594 | H-1→L+1 (79%), H→L+4 (11%), H-2→L (4%), H→L (3%) |

MO=molecular orbital, H=HOMO, L=LUMO, *f=* oscillator strength

**Table S20**: Wavelength, excitation energy and oscillator strength of **TPD5** in chloroform

| **NO** | **DFT λ (nm)** | **E(eV)** | ***f*** | **MO contributions** |
| --- | --- | --- | --- | --- |
| 1 | 1190.324 | 1.042 | 0.001 | H-3→L (41%), H-3→L+1 (45%), H-3→L+2 (5%), H-3→L+3 (3%), H-3→L+5 (4%), H-3→L+6 (3%) |
| 2 | 1189.183 | 1.043 | 0.002 | H-2→L (43%), H-2→L+1 (42%), H-2→L+2 (5%), H-2→L+3 (3%), H-2→L+5 (3%), H-2→L+6 (4%) |
| 3 | 990.922 | 1.251 | 0.991 | H→L (97%), |
| 4 | 944.498 | 1.313 | 0.000 | H→L+1 (98%), |
| 5 | 714.565 | 1.735 | 1.544 | H→L+2 (96%), H-1→L+3 (3%) |
| 6 | 653.305 | 1.898 | 0.000 | H→L+3 (96%), |

MO=molecular orbital, H=HOMO, L=LUMO, *f=* oscillator strength

**Table S21**: Wavelength, excitation energy and oscillator strength of **TPD6** in chloroform

| **NO** | **DFT λ (nm)** | **E(eV)** | ***f*** | **MO contributions** |
| --- | --- | --- | --- | --- |
| 1 | 748.652 | 1.656 | 2.056 | H→L (95%), H-1→L+1 (3%) |
| 2 | 677.065 | 1.831 | 0.070 | H→L+1 (97%), |
| 3 | 516.622 | 2.400 | 0.537 | H→L+2 (83%), H-1→L+1 (5%), H-1→L+3 (3%), H→L+4 (4%) |
| 4 | 512.416 | 2.420 | 0.075 | H-1→L (77%), H→L+3 (13%), |
| 5 | 502.204 | 2.469 | 0.021 | H-1→L (15%), H→L+3 (80%), H-1→L+2 (3%) |
| 6 | 487.819 | 2.542 | 0.298 | H-1→L+1 (69%), H→L+2 (10%), H→L+4 (11%), H-4→L (2%), H→L (3%) |

MO=molecular orbital, H=HOMO, L=LUMO, *f=* oscillator strength

| 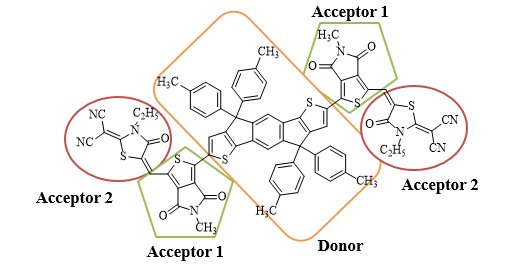 |
| --- |
| **(a)** |
| 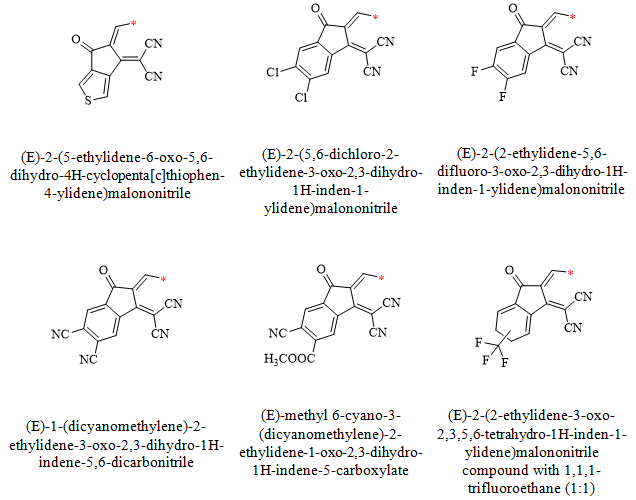 |
| **(b)** |
| **Figure S1 (a):** The schematic representation of entitled compounds. **(b):** The IUPAC names of Acceptor 2 and their structural representation |

**IUPAC names of investigated molecules**

2,2'-((5Z,5'Z)-(((4,4,9,9-tetra-p-tolyl-4,9-dihydro-s-indaceno[1,2-b:5,6-b']dithiophene-2,7-diyl)bis(5-methyl-4,6-dioxo-5,6-dihydro-4H-thieno[3,4-c] pyrrole-3,1diyl))bis (methaneylylidene))bis(6-oxo-5,6-dihydro-4H-cyclopenta[c]thiophene-5,4-diyli idene))dimalononitrile (**TPD1)**, 2,2'-((2Z,2'Z)-(((4,4,9,9-tetra-p-tolyl-4,9-dihydro-s-indaceno[1,2-b:5,6-b']dithiophene-2,7-diyl)bis(5-methyl-4,6-dioxo-5,6-dihydro-4H-thieno[3,4-c]pyrrole-3,1-diyl) )bis(methaneyly-lidene))bis(5,6-dichloro-3-oxo-2,3-dihydro-1H-indene-2,1-diylidene))dimalono- nitrile (**TPD2)**, 2,2'-((2Z,2'Z)-(((4,4,9,9-tetra-p-tolyl-4,9-dihydro-s-indaceno[1,2-b:5,6-b']dithio phene-2,7-diyl)bis(5-methyl-4,6-dioxo-5,6-dihydro-4H-thieno[3,4-c]pyrrole-3,1-diyl))bis(methane ylylidene))bis(5,6-difluoro-3-oxo-2,3-dihydro-1H-indene-2,1-diylidene))dimalononitrile (**TPD3)**, (2Z,2'Z)-2,2'-(((4,4,9,9-tetra-p-tolyl-4,9-dihydro-s-indaceno[1,2-b:5,6-b']dithiophene-2,7-diyl)bis(5-methyl-4,6-dioxo-5,6-dihydro-4H-thieno[3,4-c]pyrrole-3,1-diyl))bis(methaneylylidene))bis(1-(dicyanomethyl ene)-3-oxo-2,3-dihydro-1H-indene-5,6-dicarbonitrile) (**TPD4)**, methyl (Z)-6-cyano-2-((3-(7-(3-(((Z)-5-cyano-1-(dicyanomethylene)-6-(methoxycarbonyl)-3-oxo-1,3-dihydro-2H-inden-2-ylidene)methyl)-5-methyl-4,6-dioxo-5,6-dihydro-4H-thieno[3,4-c]pyrrol-1-yl)-4,4,9,9-tetra-p-tolyl-4,9-dihydro-s-indaceno[1,2-b:5,6-b']dithiophen-2-yl)-5-methyl-4,6-dioxo-5,6-dihydro-4H-thieno[3,4-c]pyrrol-1-yl)methylene)-1-(dicyanomethylene)-3-oxo-2,3-dihydro-1H-indene-5-carboxylate (**TPD5)** and 2,2'-((2Z,2'Z)-(((4,4,9,9-tetra-p-tolyl-4,9-dihydro-s-indaceno[1,2-b:5,6-b']dithiophene-2,7-diyl)bis(5-methyl-4,6-dioxo-5,6-dihydro-4H-thieno[3,4-c]pyrrole-3,1-diyl)) bis(methaneylylidene))bis(3-oxo-2,3,5,6-tetrahydro-1H-indene-2,1-diylidene))dimalononitrile (**TPD6)**
